# Silica Sol–Gel Coatings for Solar Panels: Drop Friction and Particle Adhesion

**DOI:** 10.1021/acsami.5c22794

**Published:** 2026-03-09

**Authors:** Tarik Karakaya, Sa’id Albarqawi, Franziska Sabath, Azadeh Sharifi-Aghili, Emre Yavuz, Doris Vollmer

**Affiliations:** Physics at Interfaces, 28308 Max Planck Institute for Polymer Research, Ackermannweg 10, 55128 Mainz, Germany

**Keywords:** wetting, pollution, durability, contact angle, friction, global warming

## Abstract

Soiling of solar panel surfaces reduces energy output and requires freshwater for cleaning. To address this challenge transparent, hydrophobic, and easy-to-clean silica-based coatings have been fabricated. Analysis of these coatings focused mostly on contact angle measurements, while drop mobility and particle adhesion—key factors for efficient soil removal—remain underexplored. To address this gap, here organically modified silicate coatings were synthesized by mixing tetraethyl orthosilicate (TEOS) with alkyl-trimethoxysilanes (alkyl-TMS) of varying alkyl chain lengths (methyl, *n*-propyl, and *n*-hexyl) and concentrations. The films showed high optical transparency (92%) and low surface roughness (0.3–1.0 nm). Increasing alkyl-TMS content and chain length increased surface hydrophobicity, leading to advancing contact angles up to 106°. By scanning drop friction microscopy and colloidal probe AFM, we quantified and correlated drop mobility and particle adhesion. A higher alkyl-TMS content led to a reduced drop friction force and reduced particle adhesion, although, mechanical robustness also decreased. Cleaning tests confirmed that coatings with lower friction and adhesion enable more effective soil removal.

## Graphical abstract

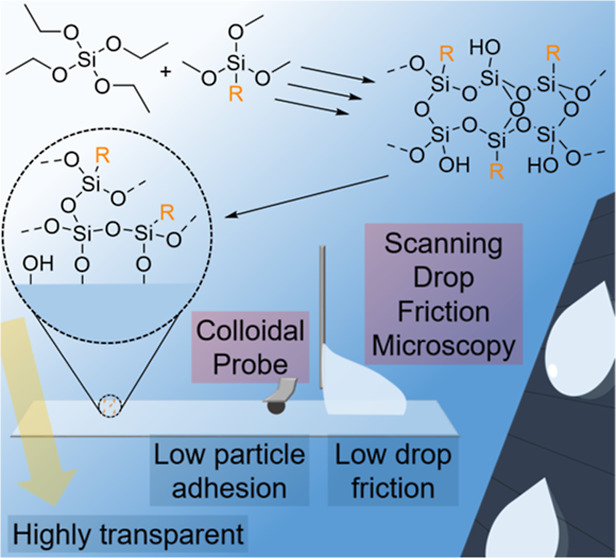

## Introduction

The sharp increase of atmospheric CO_2_ concentration has raised the global average temperature by more than 1.3 °C since the late 19th century, confronting us with growing environmental challenges.^1,2^

Therefore, it is urgent to transition to CO_2_-neutral energy sources. Energy produced from photovoltaic cells has proven to be a very promising solution.^3,4^ Over the last decades, a lot of research has been conducted to optimize the efficiency of solar cells.^5–9^ However, soiling of solar panels reduces the global photovoltaic output by at least 3–4%,^10,11^ Dust, pollen, bird droppings, and other contaminants accumulate on panel surfaces, blocking sunlight and reducing energy yield. The loss of efficiency can be overcome by cleaning, but most cleaning methods share one common drawback: a requisite for fresh water.^12^ The growing demand and increasing scarcity of fresh water worldwide underlines the urgent need for water-efficient cleaning methods.^13^ It is therefore important to develop surfaces on which water drops can easily roll off, facilitating the removal of soiling particles by taking contaminants along. Easy particle removal also requires low particle adhesion to the surface. In addition, naturally occurring water like rain or dew would further aid in passively cleaning the panels. For application on solar panel surfaces, easy-to-clean refers to surfaces where water drops can roll off with only a slight tilt, taking along contaminants because the forces driving droplet motion exceed the adhesion forces between particles and the surface. This is achieved when the surface combines low contact angle hysteresis, low drop-surface friction, and low particle adhesion, allowing passive cleaning by natural water sources such as rain or dew.^14^ For photovoltaic applications, such surfaces must additionally be transparent, mechanically robust, environmentally harmless, and cost-effective for large-scale deployment.

However, existing self-cleaning coating strategies face significant challenges. Specifically, transparent superhydrophobic coatings often lack mechanical robustness or scalability.^15,16^ This is frequently due to their reliance on rough surface structures for hydrophobicity, which are prone to degradation from mechanical friction and environmental wear, leading to poor durability and a trade-off with transparency. Furthermore, many preparation methods are high-cost, complex, or not suitable for industrial-scale production.^16,17^ On slippery lubricant-infused coatings, drops can start sliding when tilting the surface by less than 3°, but the lubricant layer depletes within a limited number of cycles.^18–22^ Fluorinated polymers exhibit high hydrophobicity, reducing particle adhesion and supporting easy sliding of water drops.^23,24^ However, due to their environmental impact and toxicity, most semifluorinated oligomers are banned or are under scrutiny for potential future restrictions.^25–27^

Among potential materials to design improved easy-to-clean coatings, silica-based coatings are a promising candidate. They can show high transparency, mechanically robustness, thermal stability, and scalability. However, pure silica-based coatings are typically hydrophilic, which promotes particle adhesion and counteracts removal of contaminants by water drops sliding-off the surface. To overcome the inherent hydrophilicity of silica, the sol–gel process, typically based on silicon alkoxides, enables the incorporation of hydrophobic moieties into the silica matrix through controlled hydrolysis and condensation reactions.^28,29^

Previous studies have employed various sol–gel techniques with silica precursors to achieve enhanced wetting properties, transparency, or reduced adhesion of particles to coated surfaces.^30–34^ The majority of these studies focus primarily on achieving improved hydrophobicity and transparency. For example, alkyl-substituted silanes have been varied to change the surface energy. However, drop friction and particle adhesion-key factors for self-cleaning of solar panels-have not been addressed quantitatively. Can drop friction and particle adhesion be reduced while still keeping the coating’s high transparency, and mechanical robustness?

We systematically investigate how varying the fraction of alkyl-trimethoxysilane (alkyl-TMS) relative to tetraethyl orthosilicate (TEOS) in mixture affects key surface properties. Using TEOS as the primary precursor, we vary both the alkyl-TMS chain length (methyl, *n*-propyl, *n*-hexyl) and the weight ratio of alkyl-TMS to TEOS (25:75, 55:45, 75:25) to tailor transparency, wetting, particle adhesion, and mechanical robustness.

Varying the chain length and concentration of alkyl-trimethoxysilanes (alkyl-TMS) in the sol–gel process allows for the tuning of surface energy. Alkyl-TMS introduces nonpolar organic groups that reduce surface energy and modify wetting behavior, while tetraethyl orthosilicate (TEOS) promotes the formation of a dense silica network. By adjusting the alkyl-TMS/TEOS ratio and the chain length of the alkyl groups, we systematically modulate coating properties relevant to self-cleaning functionality.

With increasing alkyl-TMS content and chain length, we observe an improved hydrophobicity (advancing contact angle up to 106°), reduced drop friction forces (from 55 μN for methyl-TMS down to 20 μN for *n*-hexyl-TMS), and significantly reduced particle adhesion from 0.0170 N/m to 0.0080 N/m although, mechanical robustness decreases. The coatings maintain high optical transparency (∼92%) and exhibit a smooth morphology, which is less prone to soiling.^15,35^ Drops can slide-off and take contaminants along. By combining scanning drop friction microscopy and colloidal probe AFM, this study establishes a quantitative framework to characterize both drop mobility and particle adhesion, two key yet underexplored factors for easy-to-clean solar panel coatings.

## Results and Discussion

We employed an acid-catalyzed sol–gel process, allowing precise tuning of material properties by selecting and combining different precursors, to fabricate silica-based coatings on glass substrates with targeted hydrophobicity, high transparency, and enhanced mechanical robustness. The synthesis was carried out under ambient conditions, using tetraethyl orthosilicate (TEOS) as the primary precursor to promote dense network formation and enhance hardness.^36^ Alkyl-trimethoxysilanes (alkyl-TMS) with varying alkyl chain lengths were introduced as coprecursors to modify wetting behavior. Isopropanol (IPA) was used as a solvent, and formic acid served as the acid catalyst in combination with water (Figure 1a). The process involves initial hydrolysis of Si–OR groups to form Si–OH functionalities, followed by condensation reactions that generate Si–O–Si bonds, resulting in a hybrid silica network with embedded organic moieties.

**Figure 1:**
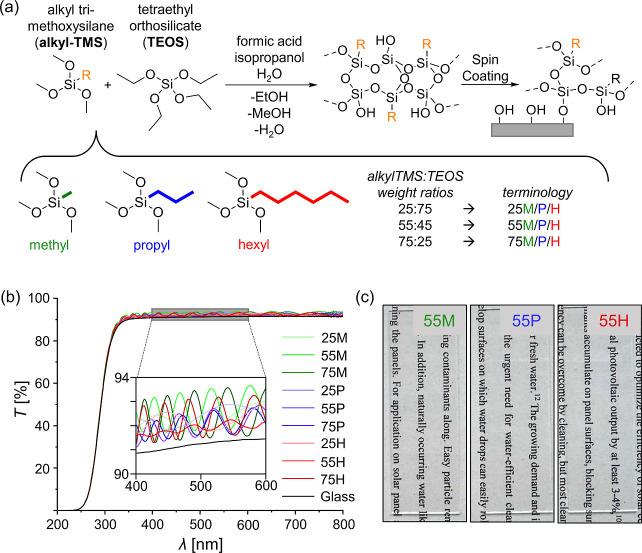
(a) Schematic illustration of the sol–gel process used to prepare silica-based coatings. The alkyl chain length was varied using methyl-TMS (M), *n*-propyl-TMS (P), and *n*-hexyl-TMS (H). Sample codes denote the alkyl-TMS content in the precursor mixture (25, 55, or 75) and the alkyl substituent (M, P, or H). (b) UV–vis transmission spectra (200–800 nm) of coated and uncoated soda-lime glass substrates. The sharp decline below 350 nm corresponds to UV absorption by the glass substrate. (c) Photographs of glass samples coated with 55M, 55P, and 55H (alkyl-TMS/TEOS = 55:45) placed over paper with printed text, demonstrating the high optical transparency of the coatings.

The solution was stirred at room temperature for 24 h to ensure sufficient hydrolysis and condensation before application. The resulting sol was then deposited via spin coating (1000 rpm, 30 s, ramp 400 rpm s^–1^) onto soda-lime glass as a model substrate for solar panel cover glass. Following deposition, the films were cured at 120 °C for 2 h.

We prepared coatings using three different alkyl-trimethoxysilanes (alkyl-TMS): methyl-TMS (M), *n*-propyl-TMS (P), and *n*-hexyl-TMS (H). In addition, the weight ratio of alkyl-TMS to TEOS was varied (25:75, 55:45, and 75:25). In the following, coatings are abbreviated by the alkyl-TMS content (25, 55, 75) followed by the letter denoting the chain length (M, P, or H). For all coatings, the sol was adjusted to a solid content of 20 wt %. In the case of 25M, 25P, and 25H, the solid content was further reduced to 10 wt % by adding additional isopropanol. This dilution ensured uniform film formation and minimized surface defects such as cracking, which occurred at higher solid contents (Figure S1).

Sample 75H required either elevated stirring temperatures or prolonged stirring to yield fully transparent coatings. When stirred for only 24 h at room temperature, the sol produced coatings that appeared clear immediately after spin-coating, but became opaque after curing due to the formation of large spherical protrusions, as revealed by AFM. In contrast, stirring the sol for 24 h at 50 °C or for 14 d at room temperature resulted in smooth coatings with no characteristic phase-separated structures. The elevated-temperature route yielded homogeneous, fine protrusions (∼4 nm height), while long-term stirring produced surfaces without notable texture. These findings highlight the sluggish hydrolysis and condensation kinetics of HTMS compared to TEOS, which led to phase separation at standard conditions. Controlled heating or extended stirring suppressed this effect and enabled transparent, homogeneous 75H coatings (Figure S2).

To enable direct comparison, we kept the synthesis and measurement conditions constant and varied only the alkyl chain (methyl, *n*-propyl, *n*-hexyl) and the alkyl-TMS/TEOS weight ratio (25:75, 55:45, 75:25). Note that the 25-series was prepared at 10 wt % solids (others 20 wt %). Characterization settings (UV–vis geometry, AFM mode/probe, contact-angle protocol, and scanning drop friction microscopy drop volume and stage speed) were also kept constant for comparability.

Transparency is the most important requisite for solar panel coatings. To quantify the transmission, all coatings and an additional uncoated glass substrate were analyzed using a UV–vis spectrometer (Cary 60, Agilent Technologies, USA) in the wavelength range of 200–800 nm (Figure 1b) referenced against air. Above 350 nm, the transmission of all coatings and the uncoated glass substrate is relatively constant. Notably, the transmission of the coated substrates (∼92%) is slightly higher than that of uncoated glass (∼91.5%).

The sharp decline of transmission at 350 nm is assigned to the absorption of UV light by the underlying glass substrate. Typical silicon-based solar cells have a band gap of 1.1 eV,^37^ corresponding to an absorption range of 400–1100 nm. Therefore, the decreased transmission below 350 nm does not affect the photovoltaic efficiency of solar panels.

Interference fringes in the spectrum of the coated samples are caused by interference between light reflected at the coating-substrate interface. This observation hints toward the homogeneous nature of the dense acid-catalyzed silica-network.^38^ By considering the maxima and minima of the refractive fringes as continuous functions of the wavelength the thickness of the coatings and their refractive indices can be determined (Figure S3).^39,40^ The coating thicknesses were 1.62 ± 0.14 μm, while the 25M, 25P, and 25H samples showed lower values of 0.52 ± 0.01 μm. This reduction results from the stronger dilution of the sols during preparation, which lowered the solid content. Figure 1c show photographs of three exemplary coatings placed over a printed text. This setup allows direct visual comparison between covered and uncovered regions. The coated glass appears fully transparent to the naked eye, as the underlying text remain clearly visible without noticeable distortion or loss in contrast. Together, these findings confirm that the alkyl-TMS coprecursors even slightly enhance the transparency of the coatings.

Since surface roughness influences the apparent wetting behavior of coatings, we quantified the root-mean-square roughness (*R*_RMS_) using atomic force microscopy (AFM, Bruker Scientific Dimension Icon, tapping mode, cantilever spring constant 26 N m^–1^). Figure 2 displays exemplary 60 × 60 μm^2^ topography images of coatings for 55 M (*R*_RMS_ = 0.30 nm), 55P (*R*_RMS_ = 0.50 nm), and 55H (*R*_RMS_ = 0.35 nm). The images reveal smooth and homogeneous surfaces. Detailed roughness and AFM data for all coatings are provided in the Supporting Information (Figure S4). The *R*_RMS_ values ranged from 0.3 to 1.0 nm. The similar *R*_RMS_ values across all coatings indicate that neither the alkyl chain length nor its concentration affected the surface roughness.

**Figure 2:**
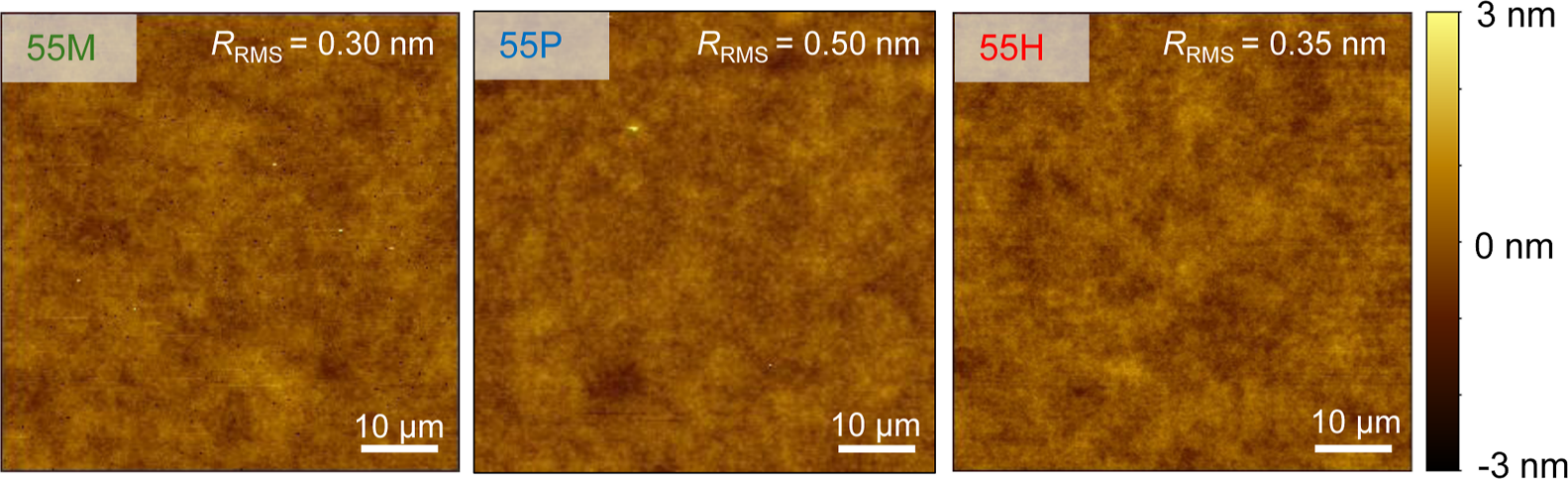
Three exemplary 60 × 60 μm^2^ AFM images of coating 55M, 55P and 55H. The root-mean-square roughness *R*_RMS_ is indicated on the top right corner of the respective AFM image. All AFM images share the same height (*z*) scale (−3 to 3 nm).

To characterize the wetting properties, advancing and receding water contact angles were determined by inflating and deflating sessile drops (5 μL) at 0.5 μL s^–1^ using a drop shape analyzing software (KRÜSS ADVANCE). The resulting contact angles are shown in Figure 3a. With increasing alkyl-TMS content (e.g., 25M < 55 M < 75M) and chain length (methyl < *n*-propyl < *n*-hexyl), both advancing and receding angles increase, while the contact angle hysteresis Δθ, defined as the difference between the advancing and the receding angles, decreases by approximately 3° between each chain length. For example, 25 M exhibits an advancing angle of 74° ± 1° with a hysteresis of 18° ± 1°, whereas 75H reaches 106° ± 3° with a hysteresis of only 10° ± 4°. These results are consistent with a reduction in surface energy which might arise from an increasing number and preferential orientation of nonpolar alkyl chains toward the coating surface.

**Figure 3:**
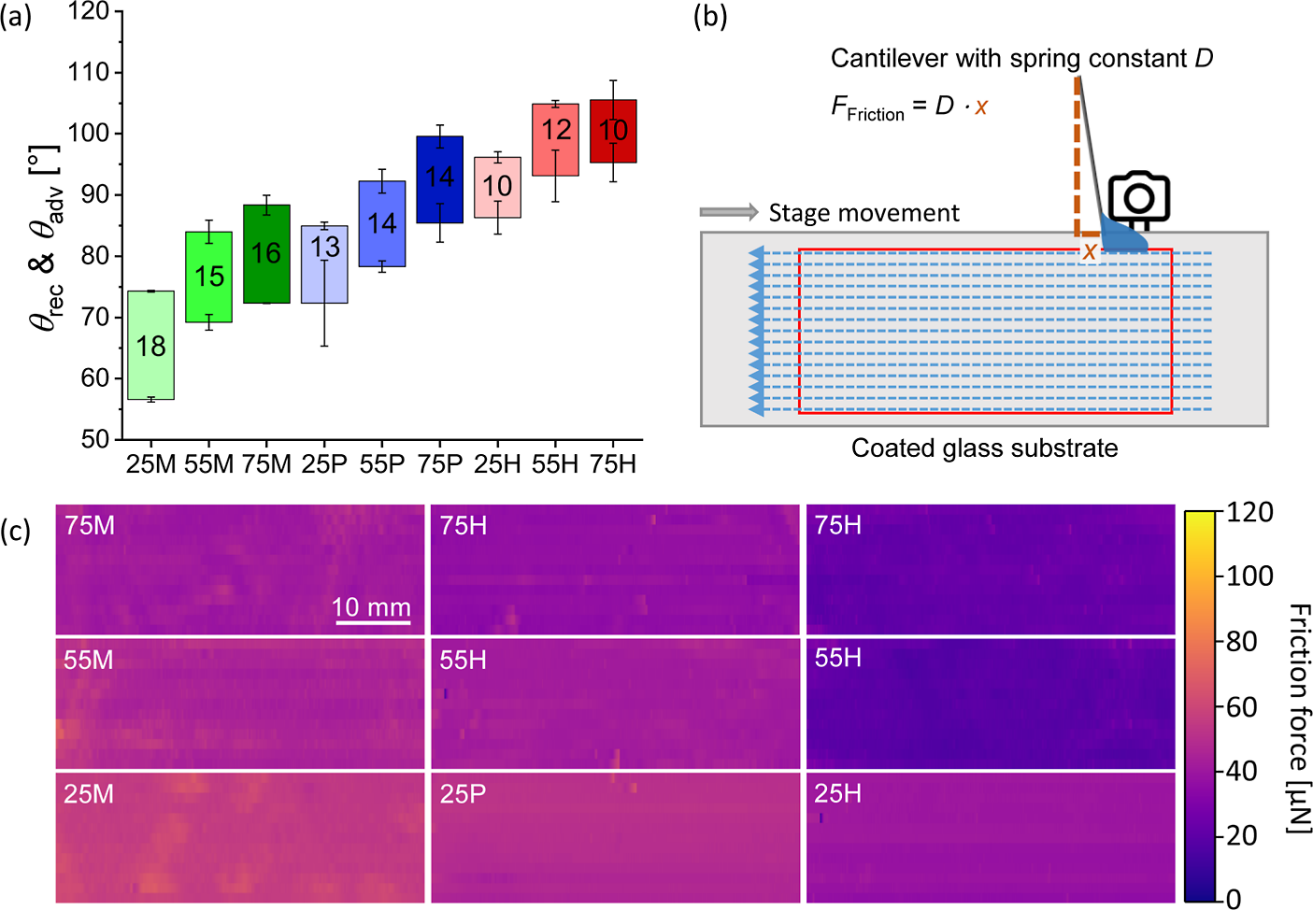
(a) Advancing (θ_adv_) and receding (θ_rec_) contact angles and the resulting contact angle hysteresis (Δθ) for coatings prepared with different alkyl-trimethoxysilane (alkyl-TMS):TEOS ratios. Sample codes indicate composition: the number (25, 55, 75) refers to the alkyl-TMS content in the precursor mixture, and the letter denotes the alkyl substituent (M = methyl-TMS, *P* = *n*-propyl-TMS, H = *n*-hexyl-TMS). (b) Schematic of the scanning drop friction force microscopy setup. A 5 μL water droplet is attached to a glass capillary and moved laterally across the coated substrate. Capillary deflection is recorded by a CMOS camera, and friction forces are calculated using Hooke’s law. (c) Friction force heat maps obtained from the kinetic regime of the lateral force measurements. Bright to dark regions represent higher to lower lateral friction forces.

To evaluate drop mobility, friction forces were measured using scanning drop friction microscopy (Figure 3b). A water drop with a fixed volume (∼5 μL) is attached to a glass capillary (spring constant *D* = 110 μN m^–1^), and the substrate is moved laterally beneath it (100 mm min^–1^). The capillary’s deflection *x* is converted to a force via Hooke’s law (*F*_*x*_ = *D*·*x*). Measurements were performed across an area of 18 × 50 mm^2^ by scanning 13 parallel lines with a spacing of 1.5 mm. Each line was recorded using a fresh water drop with an approximate diameter of 2 mm. A high-resolution image of the capillary-drop contact is shown in the Supporting Information (Figure S5). Figure 3c presents heat maps generated from the kinetic regime of the measured force curves. A decrease in friction is observed with increasing alkyl chain length and alkyl-TMS content, as reflected by the progressively darker regions in the friction force maps. Darker colors denote lower lateral forces on the water drop. For instance, the average friction force decreases from 55 ± 8 μN for 25 M to 41 ± 5 μN for 75M, and further down to 24 ± 5 μN for 75H. An exception is 55H, which shows slightly lower friction than 75H (20 ± 5 μN). The reason is not clear. Notably, no bright spots are observed across the scanned areas, which suggests the absence of localized pinning points that would otherwise indicate surface heterogeneities or defects impeding drop movement. Histograms of the measured friction forces are provided in the Supporting Information (Figure S6) to visualize the distribution and further confirm the homogeneity of the coatings.

According to the Furmidge equation^41,42^

1 $$F_{cap}\approx \gamma w\left(\cos\Theta_{rec}-\cos\Theta_{adv}\right)$$

the lateral capillary force acting on a sliding drop is governed by the surface tension γ of the liquid, the contact width *w*, and the contact angle hysteresis Δθ. Thus, both a smaller contact width and a lower hysteresis contribute to reduced friction, which is consistent with the results of the contact angle measurements showing increasing advancing and receding contact angles and decreasing hysteresis across the coating series.

No reliable friction force measurements could be obtained for the uncoated glass substrate, as the water drop adhered more strongly to the surface than to the capillary. Upon stage movement, the drop elongated, forming a persistent tail before eventually rupturing.

For water drops to take particles along, the adhesion of the particles to the surface needs to be sufficiently low. Therefore, we determined the adhesion force of our alkyl-TMS/TEOS coatings by measuring force–distance curves with the colloidal probe AFM technique. We used a Si-particle with a nominal diameter of 10 μm which was glued to a tipless cantilever (*k* = 9.96 ± 0.80 N/m) as tip and measured for each coating at least two consecutive adhesion force maps on an area of 40 × 40 μm^2^ (32 × 32 px^2^ grid) with 1024 individual force curves per map. The actual particle radius (4.7 μm) was determined using a scanning electron microscope after the measurements were taken (see Figure S7). Figure 4a shows a representative, baseline-corrected force-Δ*z* curve. Δ*z* is the *z*-piezo displacement relative to its initial position. No long-range force acts on the Si-particle, neither in the expansion region (black) nor in the retraction part (red) of the force–displacement curve.^43^ Upon contact between the coatings’ surface and Si-particle the measured force increases linearly until reaching a certain (user chosen) threshold. Thereafter, the Si-particle is withdrawn from the surface, resulting in a decrease of the measured force. Both the linear increase and decrease and the overlap of the extend and retract curves during contact between surface and Si-particle indicate that the underlying surface can be assumed as hard surface. The adhesion force between Si-particle and coating is determined as the difference between the minimum force of the linear decrease during the withdraw and the force far away from the surface. The adhesion forces for all individual force curves of each of our coatings are plotted as histograms and fitted with a Gaussian fit (see Figure S8) to determine the mean adhesion force ${\mathrm{F̅}}_{adh}$ and the error ${\Delta \mathrm{F̅}}_{adh}$ shown in Figure 4b. The Gaussian like distribution of the adhesion forces for all our coatings indicate homogeneity in case of the adhesion forces. The histogram of the coating containing 55P is comparable wide, resulting in an error twice as large as for the other alkyl-TMS/TEOS coatings (Figure S8). Neither surface inhomogeneities such as pronounced patterns nor a change of the adhesion force in the consecutive recorded maps can explain this larger error (Figure S9).

**Figure 4:**
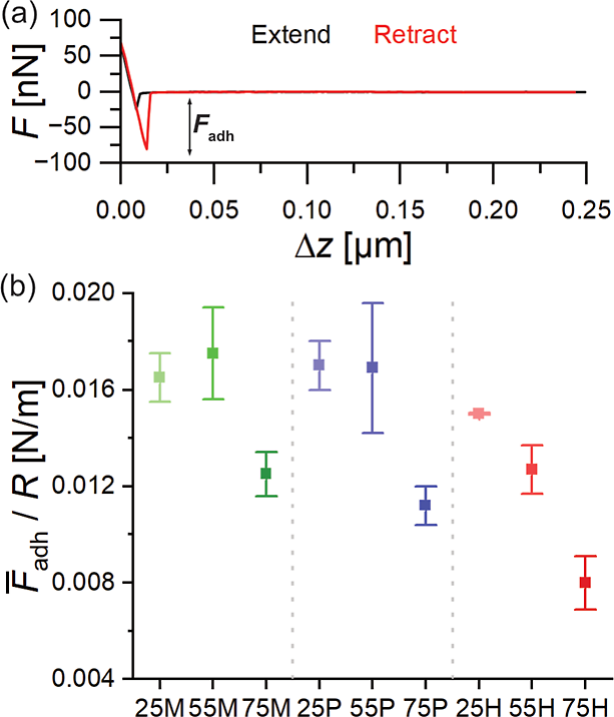
(a) A representative, baseline-corrected force-Δ*z* curve of the adhesion force between an alkyl-TMS/TEOS coating and a 10 μm Si-particle. Δ*z* is the *z*-piezo displacement relative to its initial position. The extend (black) and retract (red) curves as well as the adhesion force *F*_adh_ are highlighted. (b) Mean adhesion forces between the alkyl-TMS/TEOS coatings and the 10 μm Si-particle. Coatings containing methyl-TMS are shown in green, *n*-propyl-TMS in blue and *n*-hexyl-TMS in red, each with the different alkyl-TMS contents (25, 55, and 75). The error bars correspond to 2σ of the Gaussian fit.

Regardless of the alkyl-TMS, the coatings with the highest amount of alkyl-TMS showing always the lowest adhesion force. In case of the methyl- and *n*-propyl-TMS the mean adhesion force for the 25- and 55-series coatings lies in the range of (0.0170 ± 0.0004) N/m. By increasing the alkyl-TMS content to the 75-series, the mean adhesion force decreases to (0.0125 ± 0.0009) N/m and (0.0112 ± 0.0008) N/m, respectively. The mean adhesion forces of the coatings containing methyl- and *n*-propyl-TMS are similar. The increase in chain length from methyl to *n*-propyl is not sufficient for affecting the mean adhesion force of our coatings. The amount of methyl- and *n*-propyl-TMS only influences the mean adhesion force at the highest concentration (75-series), always showing the lowest adhesion force. For 75H it decreased by about a factor of 2 compared to 25M. Regarding the question whether the contact time between the Si-particle and underlying coating has an impact on the mean adhesion force, we also measured the mean adhesion force after 125 ms of contact (Figures S10–S12). The adhesion force differs between below 1% and 14%, typically slightly decreasing after extended contact time. The mean adhesion force of 55H coating lies in the same range as the mean adhesion forces of 75 M and 75P. It seems that the mean adhesion force is insensitive to changes in chain length in the case of methyl-to *n*-propyl-TMS. The sensitivity with which the mean adhesion force changes when the amount of alkyl-TMS is varied also appears to depend on the chain length. In terms of the easy-to-clean properties that we aim to achieve with our alkyl-TMS/TEOS coatings, the mean adhesion forces suggest that (1) particles should be easiest to remove from the 75H coating, (2) particle removal from coatings with methyl- and *n*-propyl-TMS should be similar, and (3) removing particles from coatings 75 M or 75P as well as 55H should be comparable.

The cleaning performance of selected coatings was evaluated by rinsing samples contaminated by standardized artificial soil with water.^44^ The aqueous soil mixture consisted of a 5 vol % dispersion of carbon black (1.25 g L^–1^), 47 vol % of a mineral suspension containing iron­(III) oxide, bentonite, and montmorillonite, 2 vol % of an inorganic salt solution (including sodium chloride, sodium nitrate, and calcium sulfate), and 28 vol % of a humic acid solution (1.4 g L^–1^). The mixture was applied dropwise to the coated surfaces and allowed to dry before rinsing with 4500 μL of distilled water on vertically oriented samples (90° tilt). On hydrophobic coatings, the deposited soil dries into black isolated spots. On the hydrophilic uncoated glass, in contrast, the soil solution spreads across the surface and dries into a more continuous layer. Here, a typical coffee-ring effect is visible at the edges, so the distribution is not completely homogeneous but still markedly different from the spot-like deposits on the more hydrophobic coatings. The before-and-after images show that, after applying the same amount of water evenly across the surface, a substantially greater portion of the dried soil was removed from the hexyl-modified coating compared to the methyl-modified one (Figure 5 and Video S1 and Video S2). On 55H, the water drop slide off the surface, visibly removing a large portion of the dried soil. 55 M coatings and the uncoated glass­(Video S3), by contrast, shows no cleaning effect under the same rinsing conditions. Additional experiments (Figure S13) and videos include 55P (Video S4), where the cleaning performance is intermediate: roughly half of the soil remains after allowing 6 times 750 μL of water to flow from the surface. These visual observations indicate that coatings with longer alkyl chains tend to promote better water mobility and soil removal.

**Figure 5:**
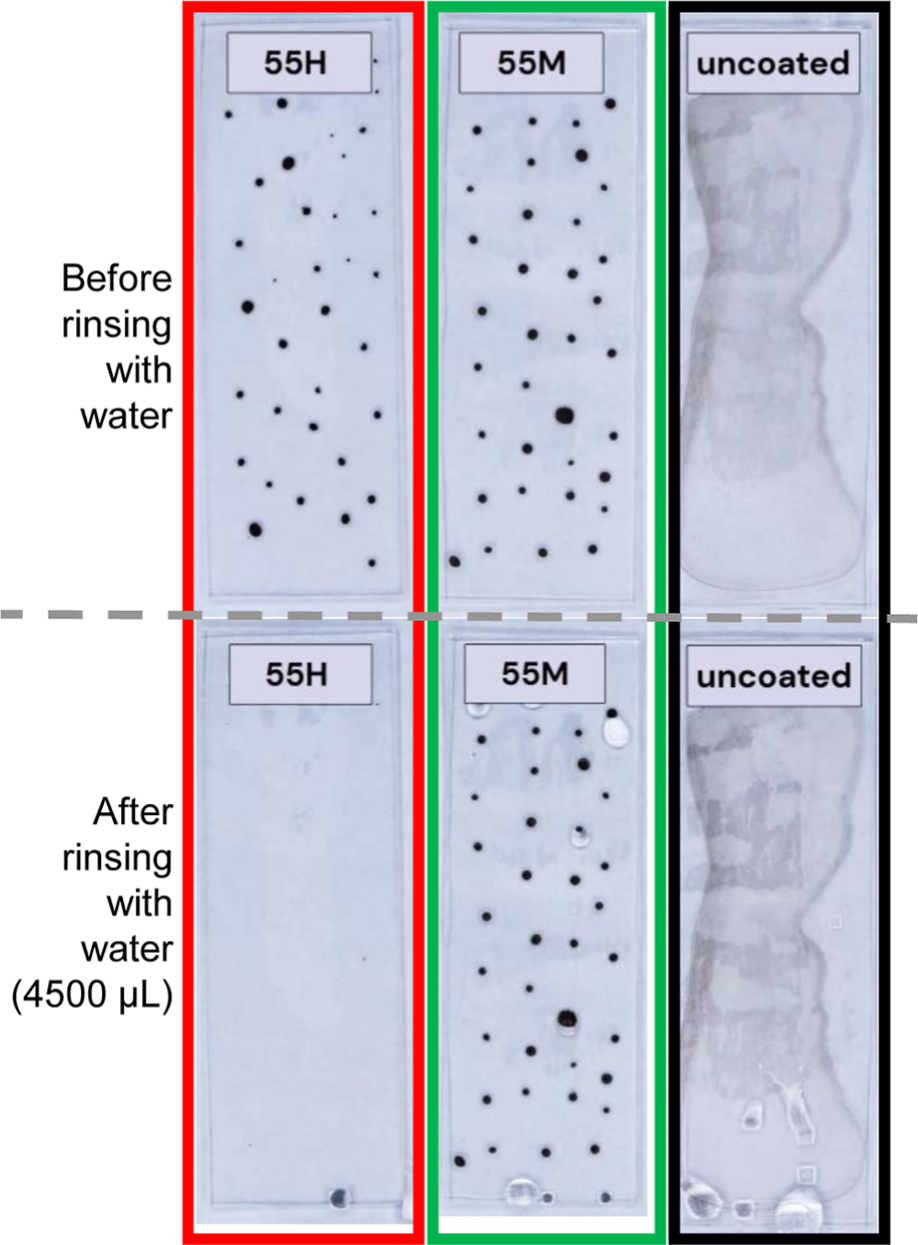
Images of coated and uncoated glass slides after soiling and rinsing experiments. Samples 55H (*n*-hexyl-TMS), 55 M (methyl-TMS), and an uncoated glass reference are shown. The upper row displays surfaces after artificial soiling (black circular spots on coated samples and a more continuous, irregularly spread soil layer on the uncoated glass). The lower row shows the same samples after rinsing with 4500 μL water. After rinsing (lower row), 55H appears completely clean by naked eye, whereas 55 M and the uncoated reference show no visible improvement.

The observed differences in cleaning behavior hint that the alkyl chains in the silica matrix orient toward the air interface during the sol–gel condensation. This outward orientation promotes water repellency, reduces particle adhesion and the interaction between the surface and soiling particles.

To assess the mechanical robustness of the coatings, two complementary measurements were performed: (1) the penetration depth while applying a normal load of up to 13 nN using atomic force microscopy (AFM), and (2) a relative scratch hardness test using a calibrated pencil method. In the AFM-based indentation experiments, a sharp silicon cantilever tip with a nominal radius of 7 nm and a spring constant of 1.4 N m^–1^ was pressed vertically into the surface while recording the cantilever deflection and *z*-piezo displacement. Multiplying the cantilever deflection d with its sensitivity and its spring constant yields in the force vs *z*-piezo displacement curve shown in Figure S14a. The indentation δ is calculated by δ = (*z* – *z*_0_) – (*d* – *d*_0_) were (*z*_0_, *d*_0_) is the contact point. We refer the maximum indentation depth as penetration depth. The force vs indentation curves for all our samples (see Figure S15) reveal a softer upper layer which is indented by the cantilever tip between 0.9 and 1.5 nm, while no indentation is observed for the harder layer underneath (see Supporting Information for more details). All coatings exhibit low penetration depths between 0.9 and 1.5 nm, independent of alkyl chain length or TMS content, Figure 6a. Young’s modulus for the upper layer for all our samples is about 10 ± 6 GPa.^45^ Such low penetration depths are beneficial for particle removal, as they minimize the mechanical embedding of contaminants and thus lower the detachment barrier during cleaning.

**Figure 6:**
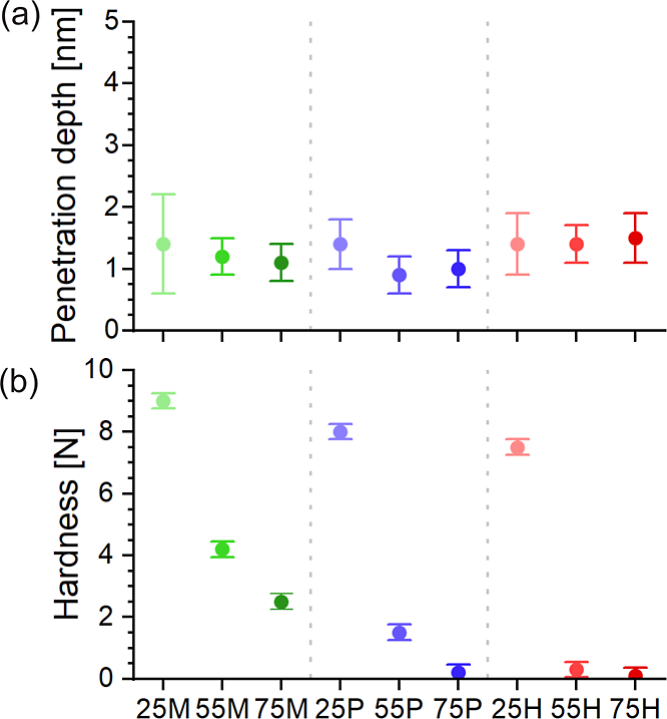
(a) Penetration depth of a cantilever with a sharp tip into the alkyl-TMS coatings and (b) hardness of the alkyl-TMS coatings. Coatings containing methyl-TMS are shown in green, *n*-propyl-TMS in blue and *n*-hexyl-TMS in red. The three different alkyl-TMS series (25, 55, and 75) are represented by a gradient from light to dark within the color mentioned. Hardness values in (b) refer to the force required to visibly scratch the surface using a pencil hardness tester, serving as a relative measure of scratch resistance.

For scratch resistance, commercial hardness pencils (Model 318S, Erichsen, Germany) were used with calibrated loads to determine the minimal force required to visibly damage the coating surface. Although the pencil test does not yield absolute hardness in units of pressure (e.g., N/mm^2^), it offers a comparative measure of scratch resistance between coatings. The scratch hardness (Figure 6b) clearly depends on both the alkyl chain length and the alkyl-TMS concentration. For all alkyl-TMS types, coatings with a lower alkyl-TMS content are significantly harder than those with higher content. For instance, the difference in scratch hardness between the low- and high-alkyl-TMS coatings (25 and 75) is 6.5 N for methyl-TMS, 7.8 N for *n*-propyl-TMS, and 7.4 N for *n*-hexyl-TMS. We attribute this trend to differences in cross-linking density: TEOS provides four cross-linking sites per molecule, while alkyl-TMS only contributes three. Increasing the alkyl-TMS fraction thus reduces the overall cross-link density and, consequently, the mechanical integrity of the network. In addition, steric hindrance introduced by longer alkyl chains (e.g., in *n*-hexyl-TMS) may inhibit the formation of dense silica networks. This effect may explain the reduced hardness at higher alkyl-TMS concentrations. Likely, the nonpolar alkyl chains are in close proximity, further reducing packing efficiency. Both, reduced cross-linking and increased steric repulsion contribute to the diminished mechanical performance. However, it is worth noting that high hardness is not always ideal for all stress scenarios. In real-world outdoor conditions, coatings must also withstand dynamic stresses such as bending or warping due to wind or thermal expansion. A slightly softer and more elastic coating might better absorb such mechanical deformations without cracking or delaminating. Thus, while high hardness improves resistance to abrasion and scratching, a balance between rigidity and flexibility may be beneficial for long-term durability.

## Summary

In this work, we present a framework to evaluate easy-to-clean silica sol–gel coatings which might prospectively be used for solar panel applications. By varying the alkyl chain length and concentration of alkyl-trimethoxysilanes (alkyl-TMS) within a TEOS-based matrix, we created coatings that combine high optical transparency (∼92%), reduced particle adhesion, and low water drop friction. The smooth surface morphology (*R*_RMS_ = 0.3–1.0 nm) ensures that differences in wetting behavior originate from surface chemistry rather than topography. To assess the easy-to-clean performance beyond dynamic contact angle measurements, we used scanning drop friction microscopy and colloidal probe AFM to quantify drop mobility and particle adhesion. Our results show a consistent trend: increasing alkyl-TMS content and chain length reduces surface energy, improves hydrophobicity, and lowers both friction and adhesion forces. For example, the coating 75H exhibited the lowest particle adhesion force ((0.0080 ± 0.0011) N/m) and drop friction (24 ± 5 μN). However, enhanced hydrophobicity comes at the cost of reduced scratch resistance due to lower cross-linking density and increased steric hindrance. This trade-off highlights the importance of balancing surface functionality and robustness. Our integrated measurement strategy enables this balance to be quantitatively assessed during material development.

## Materials and Methods

### Materials

The following chemicals were used: tetraethyl orthosilicate (TEOS, 98%; Sigma-Aldrich, China), hexyltrimethoxysilane (98%; TCI, Japan), *n*-propyltrimethoxysilane (98%; thermo scientific, Germany), methyltrimethoxysilane (97%; thermo scientific, Germany), 2-propanol (99.8%; Honeywell, Germany), formic acid (96%; Sigma-Aldrich, USA), cerium­(IV) oxide (99.9%; Aldrich,, Germany), and ethanol (99.8%; VWR Chemicals, Germany). Reagents were used as received. Soda lime glass slides of 76 × 26 × 1 mm^3^ were obtained from EprediaTM (USA). Purified Milli-Q water was used for sol–gel process, contact angle measurements and scanning drop friction microscopy.

### Preparation of Silica Sols

Reactions were carried out at ambient conditions. The respective alkyl-trimethoxysilane (methyl, propyl-, or *n*-hexyl-TMS) and tetraethyl orthosilicate (TEOS) were filled in a 20 mL glass vial at a solid weight ratio of 25:75, 55:45, or 75:25. Isopropanol (IPA) was added to adjust the total solid weight mass percentage of 20%, minimizing stress-induced cracking during drying. For formulations with an alkyl-TMS/TEOS ratio of 25:75, the total solid content was further reduced to 10 wt % by adding additional IPA to ensure uniform film formation and to prevent cracking during drying.

While stirring at 750 rpm, formic acid was added at a fixed volume ratio of 1:30 relative to the combined silane precursors, promoting homogeneous chain-like network formation and suppressing aggregation. Deionized water was added in a stoichiometric amount corresponding to the number of alkoxy groups in the precursors to ensure complete hydrolysis without inhibiting subsequent condensation. The resulting sol was stirred continuously for 24 h at ambient conditions. All parameters (IPA volume, acid/water ratios, stirring speed, and reaction time) were kept constant for all formulations unless stated otherwise.

### Preparation of Coatings

Soda-lime glass slides were mechanically polished using a cloth with cerium oxide. After polishing, the substrates were rinsed with Milli-Q water, sonicated in ethanol for 15 min, rinsed again with ethanol, and dried using compressed air. Before deposition, the silica sols were filtered through a 0.45 μm PVDF syringe filter (MILLIPORE, Germany) to remove particulates. Coatings were applied by spin-coating (SUSS Labspin 6 TT) at 1000 rpm for 30 s with an acceleration of 400 rpm s^–1^. The samples were subsequently cured in a convection oven at 120 °C for 2 h and cooled slowly to room temperature within the oven. Before each characterization or experiment, the slides were rinsed with distilled water to remove dust and possible surface contaminants.

### Transparency

To determine the optical transmittance of the coatings, a Cary 60 UV–vis spectrophotometer (Agilent Technologies, USA) equipped with Cary WinUV software was used. Measurements were performed in the wavelength range of 200–800 nm with a scan rate of 600 nm/min. Air was used as the reference for baseline correction. Uncoated and coated soda-lime glass slides were analyzed under identical conditions. The samples were placed perpendicular to the beam path, and no additional background correction was applied.

### Simulated Soiling and Cleaning Procedure

To evaluate the easy-to-clean performance, simulated soiling experiments were performed based on the protocol described by Sleiman et al.^44^ The artificial soiling mixture consisted of four components:

Five vol % of a 1.25 g L^–1^ aqueous dispersion of carbon black; 47 vol % of a aqueous mineral mixture containing 0.3 g L^–1^ iron­(III) oxide, 1 g L^–1^ bentonite, and 1 g L^–1^ montmorillonite; 2 vol % of an aqueous inorganic salt solution containing 0.3 g L^–1^ sodium chloride, 0.4 g L^–1^ sodium nitrate, and 0.4 g L^–1^ calcium sulfate; and 28 vol % of a aqueous 1.4 g L^–1^ humic acid solution.

Prior to application, the soiling mixture was thoroughly shaken to ensure homogeneity. Small drops were randomly deposited onto coating surfaces and dried in an oven at 50 °C for 30 min. Cleaning was conducted by applying 4500 μL of Milli-Q water in 750 μL steps (or 250 μL steps for 55M) onto the contaminated surface while the sample was held vertically (90° tilt), allowing the water to flow over the surface.

### Scratch Resistance

Scratch resistance was evaluated using a Hardness Test Pencil (Model 318S, Erichsen, Germany). A defined normal force was applied to the coating surface by adjusting the loading on the pencil tip. The applied force was gradually increased until a visible scratch appeared on the surface. Each measurement was repeated three times per sample to ensure reproducibility. Tests were performed on both uncoated and coated soda-lime glass substrates under ambient conditions.

### Atomic Force Microscopy

#### Topography Imaging

For surface imaging a Dimension Icon (Bruker Scientific Dimension Icon) was used in the soft tapping mode. OTESPA cantilevers (OPUS by MikroMasch), with resonance frequency of 300 kHz, spring constant of 26 N m^1–^, and length of 160 μm, were applied for all measurements. The images were recorded in 60 × 60 μm^2^ size. To correct the piezo drift, a polynomial background of third order was subtracted. The software Gwyddion was used to calculate the *R*_RMS_ values.

#### Adhesion Force Measurements

To determine the adhesion force, a commercial AFM (Nanowizard, JPK, Germany) was used to measure so-called force curves (deflection vs *z*-piezo displacement curves). Two types of cantilevers were used: Cantilevers with a sharp tip (HQ/NSC35/tipless/no Al, MikroMasch, Bulgaria) and tipless cantilevers (HQ/NSC35/tipless/Al BS Cantilever A, MikroMasch, Bulgaria), to which a silica particle with a nominal size of 10 μm (BS-Partikel GmbH, Germany) was glued. Figure S7 shows a SEM image of the particle used for all adhesion force measurements. A micromanipulator (Narishige, MMo 203, Japan) and under an optical microscope was used to attach the Si-particle to the tipless cantilever. A drop of dispersed 10 μm Si-particles was spread on a coverslip and dried in air. A small amount of epoxy resin (UHU Plus Endfest) was picked up with the cantilever mounted on the micromanipulator. Afterward, a 10 μm Si-particle was taken up with the cantilever. Once the epoxy resin has dried overnight, the cantilever can be used for measuring the adhesion force. Regardless of the cantilever type, the force curves where measured automatically over a grid of 32 × 32 px^2^. The measuring area was 1 × 1 μm^2^ for the cantilever with the sharp tip and 40 × 40 μm^2^ cantilever with the Si-particle. The force curves were recorded with a *z-*length of 250 nm, a *z*-velocity of 2 μm/s, and a *z*-resolution of 0.1 nm/px and 1 nm/px for the cantilever with the sharp tip and the cantilever with the Si-particle, respectively. For the cantilever with the Si particles, contact times at the sample surface of 0 and 125 ms were chosen. The measured deflection is multiplied by the sensitivity of the cantilever and the spring constant to obtain the force. The sensitivity was determined using the linear increase of the contact area of the force curves and the spring constant using the thermal method.^43,46^ A self-written program was used to evaluate the force curves. The following procedure is used to determine the adhesion force: (1) the noncontact area of the force curves was fitted with a straight line, (2) the minimum of the contact area (“jump-out contact point”) of the retracting force curve was determined and (3) the difference between these two values gives the adhesion force. The adhesion forces were displayed in a histogram and fitted with a Gaussian fit, which gives the mean adhesion force *F̲*_adh_ and the error Δ*F*_adh_.

### Contact Angle Measurements

Dynamic contact angle measurements were performed using a goniometer (KRÜSS, Germany) equipped with KRÜSS ADVANCE software. To determine advancing and receding contact angles (θ_adv_ and θ_rec_), a drop of distilled water (3 μL) was deposited onto the sample surface using a Hamilton syringe fitted with a PTFE-coated needle. The drop was subsequently inflated to 40 μL and deflated back to its original volume at a constant flow rate of 0.5 μL s^–1^. Each measurement was repeated at three different positions per sample, and every cycle was performed three times to ensure reproducibility. Contact angles were determined by elliptical fitting of the drop shape. The advancing and receding angles were averaged across all measurements, and the contact angle hysteresis Δθ was calculated as the difference between θ_adv_ and θ_rec_.

### Scanning Drop Friction Microscopy

Drop friction forces were quantified using scanning drop friction microscopy as described previously in the literature.^47^ A glass capillary (50 × 0.5 × 0.05 mm^3^) with a known spring constant of 110 μN mm^–1^ was positioned approximately 1 mm above the coated surface. A 5 μL drop of Milli-Q water was placed at the tip of the capillary, allowing it to adhere. The sample stage was then moved laterally (*x*-direction) over a distance of 60 mm at a speed of 100 mm min^–1^, while the capillary deflection was recorded using a CMOS camera at 30 fps and ∼100 px mm^–1^ spatial resolution. The capillary deflection was analyzed frame-by-frame using custom software, and the deflection values were converted into lateral friction forces via Hooke’s law. The scanning was performed over a defined central area of 18 × 60 mm^2^ by executing 13 parallel lines spaced 1.5 mm apart. After each scan line, the capillary was retracted to detach from the drop and repositioned. For data analysis, the first and last 5 mm of each track were excluded to ensure evaluation of cantilever deflection within the steady-state sliding regime. The resulting data set was used to generate spatially resolved 2D heat maps of the friction forces.

## Supplementary Material











## Notes

Crack formation in 25 wt % coatings at lower dilution; effect of sol-aging on 75H coating homogeneity; UV–vis transmission analysis for refractive index and coating thickness; AFM surface topography and roughness; scanning drop friction traces to friction heatmaps and histograms; colloidal probe AFM particle size and adhesion force measurements; analysis of 55P adhesion; adhesion measurements at extended contact times; and image series of the cleaning experiments (PDF).
Supporting Videos S1–S4. Rinsing procedure used for cleaning: comparison of 55H, 55P, 55M, and uncoated glass surfaces (MP4)
(MP4)
(MP4)
(MP4)

## †

Institute for Photovoltaics, University of Stuttgart, Pfaffenwaldring 47, 70569 Stuttgart, Germany

## ‡

Carl Zeiss Vision GmbH, ZEISS Vision Care, Turnstrasse 27, 73430 Aalen, Germany.

## Abbreviations

TEOS: tetraethyl orthosilicate
TMS: trimethoxysilane
*R*_RMS_: root-mean-square roughness
AFM: atomic force microscopy
UV–vis: ultraviolet–visible
IPA: isopropanol
PVDF: poly­(vinylidene fluoride)
SEM: scanning electron microscopy
SI: supporting information
γ: surface tension
Δθ: contact angle hysteresis

## References

[1] Lindsey, R. ; Dahlman, L. Climate Change: Global Temperature; NOAA Climate.gov: Washington D.C., 2024. https://www.climate.gov/news-features/understanding-climate/climate-change-global-temperature (accessed January 9, 2025).

[2] Gibbens, S. How Global Warming Is Disrupting Life on Earth; National Geographic 2024.

[3] Wang F., Harindintwali J. D., Yuan Z., Wang M., Wang F., Li S., Yin Z., Huang L., Fu Y., Li L., Chang S. X., Zhang L., Rinklebe J., Yuan Z., Zhu Q., Xiang L., Tsang D. C. W., Xu L., Jiang X., Liu J., Wei N., Kästner M., Zou Y., Ok Y. S., Shen J., Peng D., Zhang W., Barceló D., Zhou Y., Bai Z., Li B., Zhang B., Wei K., Cao H., Tan Z., Zhao L., He X., Zheng J., Bolan N., Liu X., Huang C., Dietmann S., Luo M., Sun N., Gong J., Gong Y., Brahushi F., Zhang T., Xiao C., Li X., Chen W., Jiao N., Lehmann J., Zhu Y.-G., Jin H., Schäffer A., Tiedje J. M., Chen J. M. (2021). Technologies
and Perspectives for Achieving Carbon Neutrality. Innovation.

[4] Obaideen K., Olabi A. G., Al Swailmeen Y., Shehata N., Abdelkareem M. A., Alami A. H., Rodriguez C., Sayed E. T. (2023). Solar Energy: Applications,
Trends Analysis, Bibliometric Analysis and Research Contribution to
Sustainable Development Goals (SDGs). Sustainability.

[5] Shubbak M. H. (2019). Advances
in Solar Photovoltaics: Technology Review and Patent Trends. Renew. Sustain. Energy Rev..

[6] Almosni S., Delamarre A., Jehl Z., Suchet D., Cojocaru L., Giteau M., Behaghel B., Julian A., Ibrahim C., Tatry L., Wang H., Kubo T., Uchida S., Segawa H., Miyashita N., Tamaki R., Shoji Y., Yoshida K., Ahsan N., Watanabe K., Inoue T., Sugiyama M., Nakano Y., Hamamura T., Toupance T., Olivier C., Chambon S., Vignau L., Geffroy C., Cloutet E., Hadziioannou G., Cavassilas N., Rale P., Cattoni A., Collin S., Gibelli F., Paire M., Lombez L., Aureau D., Bouttemy M., Etcheberry A., Okada Y., Guillemoles J.-F. (2018). Material
Challenges for Solar Cells in the Twenty-First Century: Directions
in Emerging Technologies. Sci. Technol. Adv.
Mater..

[7] Giannouli M. (2021). Current Status
of Emerging PV Technologies: A Comparative Study of Dye-Sensitized,
Organic, and Perovskite Solar Cells. Int. J.
Photoenergy.

[8] Pastuszak J., Węgierek P. (2022). Photovoltaic
Cell Generations and Current Research
Directions for Their Development. Materials.

[9] Nayak P. K., Mahesh S., Snaith H. J., Cahen D. (2019). Photovoltaic Solar
Cell Technologies: Analysing the State of the Art. Nat. Rev. Mater..

[10] Ilse K., Micheli L., Figgis B. W., Lange K., Daßler D., Hanifi H., Wolfertstetter F., Naumann V., Hagendorf C., Gottschalg R., Bagdahn J. (2019). Techno-Economic Assessment of Soiling
Losses and Mitigation Strategies for Solar Power Generation. Joule.

[11] Maghami M. R., Hizam H., Gomes C., Radzi M. A., Rezadad M. I., Hajighorbani S. (2016). Power Loss Due to Soiling on Solar Panel: A Review. Renew. Sustain. Energy Rev..

[12] Jamil W. J., Abdul Rahman H., Shaari S., Salam Z. (2017). Performance
Degradation
of Photovoltaic Power System: Review on Mitigation Methods. Renew. Sustain. Energy Rev..

[13] Water for Prosperity and Peace; UN Water The United Nations World Water Development Report; UNESCO: Paris, 2024.

[14] Geyer F., D’Acunzi M., Sharifi-Aghili A., Saal A., Gao N., Kaltbeitzel A., Sloot T.-F., Berger R., Butt H.-J., Vollmer D. (2020). When and How
Self-Cleaning of Superhydrophobic Surfaces
Works. Sci. Adv..

[15] Verho T., Bower C., Andrew P., Franssila S., Ikkala O., Ras R. H. A. (2011). Mechanically Durable Superhydrophobic
Surfaces. Adv. Mater..

[16] Ingole S. S., Sutar R. S., Gaikwad P. P., Jundle A. R., Ekunde R. A., Liu S., Latthe S. S. (2025). A Review on Transparent
Superhydrophobic Coatings for
Self-Cleaning Solar Cell Panels: Its Fabrication, Robustness and Industrial
Implementation. Surf. Interfaces.

[17] Guo R., Wang Y., Lu H., Wang S., Wang B., Zhang Q. (2024). Micron-Smooth, Robust Hydrophobic Coating for Photovoltaic Panel
Surfaces in Arid and Dusty Areas. Coatings.

[18] Naga A., Scarratt L. R. J., Neto C., Papadopoulos P., Vollmer D. (2025). Drop Friction and Failure on Superhydrophobic and Slippery
Surfaces. ACS Nano.

[19] Schellenberger F., Xie J., Encinas N., Hardy A., Klapper M., Papadopoulos P., Butt H.-J., Vollmer D. (2015). Direct Observation
of Drops on Slippery
Lubricant-Infused Surfaces. Soft Matter.

[20] Baumli P., D’Acunzi M., Hegner K. I., Naga A., Wong W. S. Y., Butt H.-J., Vollmer D. (2021). The Challenge of Lubricant-Replenishment
on Lubricant-Impregnated Surfaces. Adv. Colloid
Interface Sci..

[21] Kreder M. J., Daniel D., Tetreault A., Cao Z., Lemaire B., Timonen J. V. I., Aizenberg J. (2018). Film Dynamics
and Lubricant Depletion
by Droplets Moving on Lubricated Surfaces. Phys.
Rev. X.

[22] Smith J. D., Dhiman R., Anand S., Reza-Garduno E., Cohen R. E., McKinley G. H., Varanasi K. K. (2013). Droplet
Mobility
on Lubricant-Impregnated Surfaces. Soft Matter.

[23] Cherupurakal N., Mozumder M. S., Mourad A.-H. I., Lalwani S. (2021). Recent Advances in
Superhydrophobic Polymers for Antireflective Self-Cleaning Solar Panels. Renew. Sustain. Energy Rev..

[24] Adak D., Bhattacharyya R., Barshilia H. C. (2022). A State-of-the-Art Review on the
Multifunctional Self-Cleaning Nanostructured Coatings for PV Panels,
CSP Mirrors and Related Solar Devices. Renew.
Sustain. Energy Rev..

[25] Mozumder M. S., Mourad A.-H. I., Pervez H., Surkatti R. (2019). Recent Developments
in Multifunctional Coatings for Solar Panel Applications: A Review. Sol. Energy Mater. Sol. Cells.

[26] Gerardo, O.-B. L. ; Paola, B.-C. A. Risk Assessment and Impact of Fluoride and Perfluorocarbons. In Fluoride and Fluorocarbon Toxicity; Kumar, N. , Ed.; Environmental Science and Engineering; Springer Nature Singapore: Singapore, 2024; pp 163–189.

[27] Per- and Polyfluoroalkyl Substances (PFAS); European Chemical Agency: Helsinki, Finland. https://echa.europa.eu/hot-topics/perfluoroalkyl-chemicals-pfas (accessed January 9, 2025).

[28] Hench L. L., West J. K. (1990). The Sol-Gel Process. Chem. Rev..

[29] Syafiq A., Pandey A. K., Adzman N. N., Rahim N. A. (2018). Advances in Approaches
and Methods for Self-Cleaning of Solar Photovoltaic Panels. Sol. Energy.

[30] Venkateswara
Rao A., Latthe S. S., Nadargi D. Y., Hirashima H., Ganesan V. (2009). Preparation of MTMS Based Transparent Superhydrophobic
Silica Films by Sol–Gel Method. J. Colloid
Interface Sci..

[31] Vidal K., Gómez E., Goitandia A. M., Angulo-Ibáñez A., Aranzabe E. (2019). The Synthesis of a
Superhydrophobic and Thermal Stable
Silica Coating via Sol-Gel Process. Coatings.

[32] Mahadik S. A., Mahadik D. B., Parale V. G., Wagh P. B., Gupta S., Venkateswara Rao A. (2012). Recoverable and Thermally Stable
Superhydrophobic Silica
Coating. J. Sol-Gel Sci. Technol..

[33] Cai S., Zhang Y., Zhang H., Yan H., Lv H., Jiang B. (2014). Sol–Gel Preparation of Hydrophobic
Silica Antireflective Coatings
with Low Refractive Index by Base/Acid Two-Step Catalysis. ACS Appl. Mater. Interfaces.

[34] Latthe S. S., Imai H., Ganesan V., Rao A. V. (2009). Superhydrophobic
Silica Films by Sol–Gel Co-Precursor Method. Appl. Surf. Sci..

[35] Sarver T., Al-Qaraghuli A., Kazmerski L. L. (2013). A Comprehensive
Review of the Impact
of Dust on the Use of Solar Energy: History, Investigations, Results,
Literature, and Mitigation Approaches. Renew.
Sustain. Energy Rev..

[36] Hu L., Zhang X., Sun Y., Williams R. J. J. (2005). Hardness and
Elastic Modulus Profiles of Hybrid Coatings. J. Sol-Gel Sci. Technol..

[37] Battaglia C., Cuevas A., De Wolf S. (2016). High-Efficiency Crystalline
Silicon
Solar Cells: Status and Perspectives. Energy
Environ. Sci..

[38] Vincent A., Babu S., Brinley E., Karakoti A., Deshpande S., Seal S. (2007). Role of Catalyst on
Refractive Index Tunability of Porous Silica
Antireflective Coatings by Sol–Gel Technique. J. Phys. Chem. C.

[39] Swanepoel R. (1983). Determination
of the Thickness and Optical Constants of Amorphous Silicon. J. Phys. E: Sci. Instrum..

[40] Manifacier J. C., Gasiot J., Fillard J. P. (1976). A Simple Method
for the Determination
of the Optical Constants n, k and the Thickness of a Weakly Absorbing
Thin Film. J. Phys. E: Sci. Instrum..

[41] Furmidge C. G. L. (1962). Studies
at Phase Interfaces. I. The Sliding of Liquid Drops on Solid Surfaces
and a Theory for Spray Retention. J. Colloid
Sci..

[42] Kawasaki K. (1960). Study of Wettability
of Polymers by Sliding of Water Drop. J. Colloid
Sci..

[43] Butt H.-J., Cappella B., Kappl M. (2005). Force Measurements
with the Atomic
Force Microscope: Technique, Interpretation and Applications. Surf. Sci. Rep..

[44] Sleiman M., Kirchstetter T. W., Berdahl P., Gilbert H. E., Quelen S., Marlot L., Preble C. V., Chen S., Montalbano A., Rosseler O., Akbari H., Levinson R., Destaillats H. (2014). Soiling of
Building Envelope Surfaces and Its Effect on Solar Reflectance –
Part II: Development of an Accelerated Aging Method for Roofing Materials. Sol. Energy Mater. Sol. Cells.

[45] Lima I. V. M., Silva A. V. S., Sousa F. D., Ferreira W. P., Freire R. S., De Oliveira C. L. N., De Sousa J. S. (2024). Measuring the Viscoelastic
Relaxation
Function of Cells with a Time-Dependent Interpretation of the Hertz-Sneddon
Indentation Model. Heliyon.

[46] Butt H.-J., Jaschke M. (1995). Calculation of Thermal
Noise in Atomic Force Microscopy. Nanotechnology.

[47] Hinduja C., Laroche A., Shumaly S., Wang Y., Vollmer D., Butt H.-J., Berger R. (2022). Scanning Drop
Friction Force Microscopy. Langmuir.

