# Supplementary material for: Silica Sol–Gel Coatings for Solar Panels: Drop Friction and Particle Adhesion
Source: ACS Appl Mater Interfaces. 2026 Mar 9;18(11):17073–82. doi: 10.1021/acsami.5c22794 (PMC13022817; doi:10.1021/acsami.5c22794)
Supplement: Supplementary file 1 [file am5c22794_si_001.pdf]

# Silica Sol-Gel Coatings for Solar Panels: Drop Friction and Particle Adhesion

*Tarik Karakaya[1‡], Sa'id Albarqawi[1‡], Franziska Sabath[1], Azadeh Sharifi – Aghili[1],*

*Emre Yavuz[1], Doris Vollmer[1]**

[1]Physics at Interfaces, Max Planck Institute for Polymer Research, Ackermannweg 10, 55128 Mainz, Germany

*corresponding author's email address: vollmerd@mpip-mainz.mpg.de

# Crack Formation

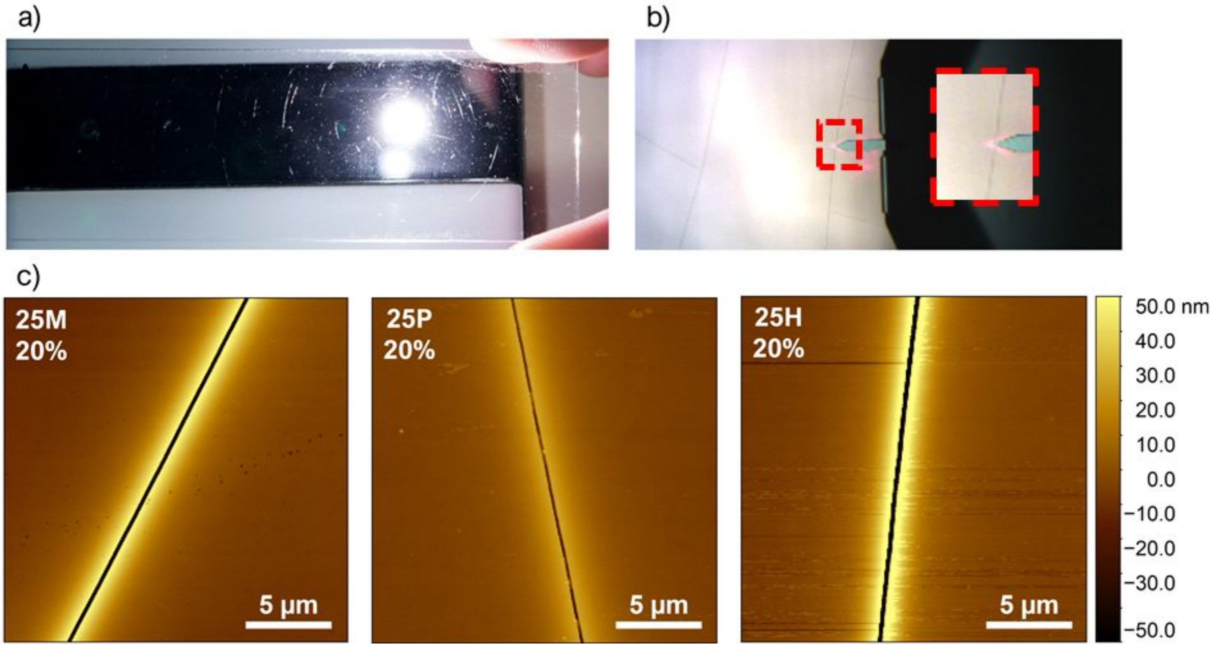


**Figure S1.** Cracks in the 25-series alkyl-TMS coatings at 20% dilution. a) 25H held over a flashlight showcasing cracks going through the sample. b) The cracks at 50x magnification. c) Cracks on 25M, 25P, and 25H at 20% dilution imaged with soft tapping mode AFM.

Thermal curing of the 25-series alkyl-TMS coatings at 20% precursor solids content resulted in the formation of distinct cracks visible to the naked eye when the samples were held against a light source (**Figure S1a**). Optical microscopy at 50× magnification (**Figure S1b**) reveals that these cracks extend continuously across the coating surface. Atomic force microscopy (soft tapping mode) of representative regions on 25M, 25P, and 25H coatings (**Figure S1c**) confirms that the cracks are several hundred nanometers wide (≈ 300–350 nm) with raised edges protruding 20–40 nm above the surrounding surface. The uniform crack morphology across all three alkyl chain lengths suggests that the substituent chemistry plays a minor role in this failure mode. Instead, the cracking can be attributed to the higher brittleness of TEOS-rich compositions, which form a densely cross-linked inorganic network with low elasticity. During solvent evaporation and subsequent cooling after curing at 120 °C, the coatings are unable to dissipate the mechanical stress through elastic deformation, resulting in fracture formation. Reducing the solids content from 20% to 10% effectively eliminated visible cracking, as the resulting thinner films remain below the critical thickness for crack propagation under the applied thermal curing conditions.

# 75H Coating behavior

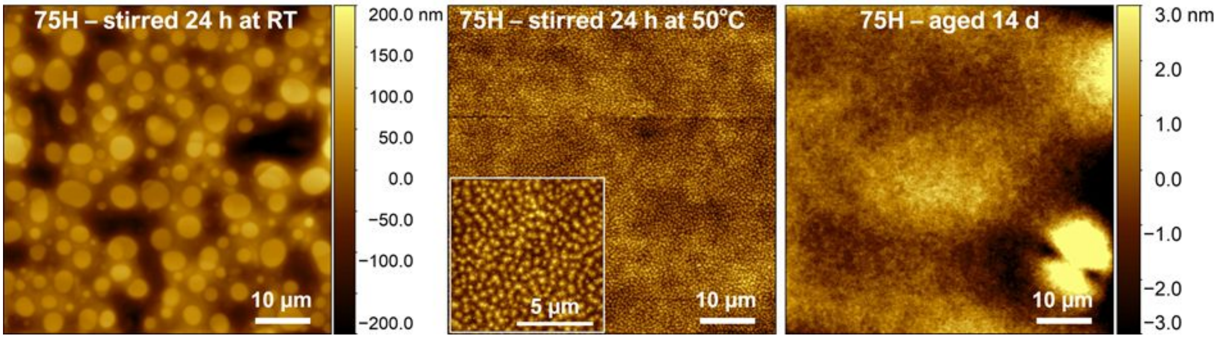


**Figure S2.** AFM images of the 75H coated glass slides with different sol stirring conditions. The sol stirring conditions are labeled onto each resulting surface image. The image of 75H stirred for 14 d and 75H stirred at 50 °C share the same height (z) scale of -3 - 3 nm.

For sample 75H, transparent coatings were only obtained after either extended stirring or elevated stirring temperature. Stirring for 24 h at room temperature led to coatings that appeared transparent after spin-coating but became opaque after curing, which AFM revealed to result from large spherical protrusions. In contrast, stirring for 24 h at 50 °C produced a fine, homogeneous surface with protrusions of only a few nanometers, while stirring for 14 d at room temperature yielded smooth films without characteristic surface features. These results indicate that the sluggish hydrolysis and condensation kinetics of HTMS compared to TEOS promote phase separation under standard conditions, which can be suppressed by controlled heating or prolonged stirring.

# Optical Thickness and Refractive Index Evaluation

The optical properties of the coatings were characterized by recording their UV–vis transmission spectra in the wavelength range of 200–800 nm (1 nm interval) using a spectrophotometer (Cary 60, Agilent Technologies). Air served as the reference. All coated samples exhibited high transparency in the visible range (≈ 92% between 400–800 nm), comparable to or slightly exceeding that of bare glass. A pronounced absorption edge near 300 nm is attributed to the substrate. Interference fringes in the transmission spectra, caused by multiple reflections at the air–coating and coating–substrate interfaces, were used to determine the refractive index and optical thickness of the films. Data were smoothed using a Savitzky–Golay filter, and the upper

and lower envelope curves were fitted numerically. The refractive indices $n_c$ were calculated following the methods in ref. [1] and [2]:

$$n_c = \sqrt{N + \sqrt{N^2 - {n_o}^2 {n_s}^2}} \qquad (1)$$

With

$$N = \frac{{n_0}^2 + {n_S}^2}{2} + 2n_0 n_S \frac{\tau_{max} - \tau_{min}}{\tau_{max}\tau_{min}} \qquad (2)$$

where $n_0$ is the refractive index of air (1.00), $n_s$ is the refractive index of the glass substrate (1.51), and $\tau_{max}$ and $\tau_{min}$ are the transmittance values at the envelope maxima and minima, respectively. Film thickness $d$ was determined from the wavelength positions ($\lambda_1$, $\lambda_2$) of two successive interference maxima (or minima) using:

$$d = \frac{M\lambda_1\lambda_2}{2(n_c(\lambda_1)\lambda_2 - n_c(\lambda_2)\lambda_1)} \qquad (3)$$

where $M$ is the number of oscillations between $\lambda_1$ and $\lambda_2$. The results (**Table S1**) show refractive indices between 1.53 and 1.59 for all coatings, with minimal wavelength dependence, indicating comparable internal pore structure across all formulations. Thicknesses of 0.51 ± 0.01 µm for the 25-series alkyl-TMS coatings(diluted to 10% solids) and 1.62 ± 0.14 µm for the 55- and 75-series coatings were determined. The thinner films in the 25-series are consistent with reduced solids content during deposition. Slight deviations (e.g., 55P and 75H) are likely due to viscosity differences in the sols during spin coating. Overall, the comparable refractive indices and high transmittance confirm that variations in wetting, friction, and adhesion behavior are not due to significant differences in optical density or transparency.

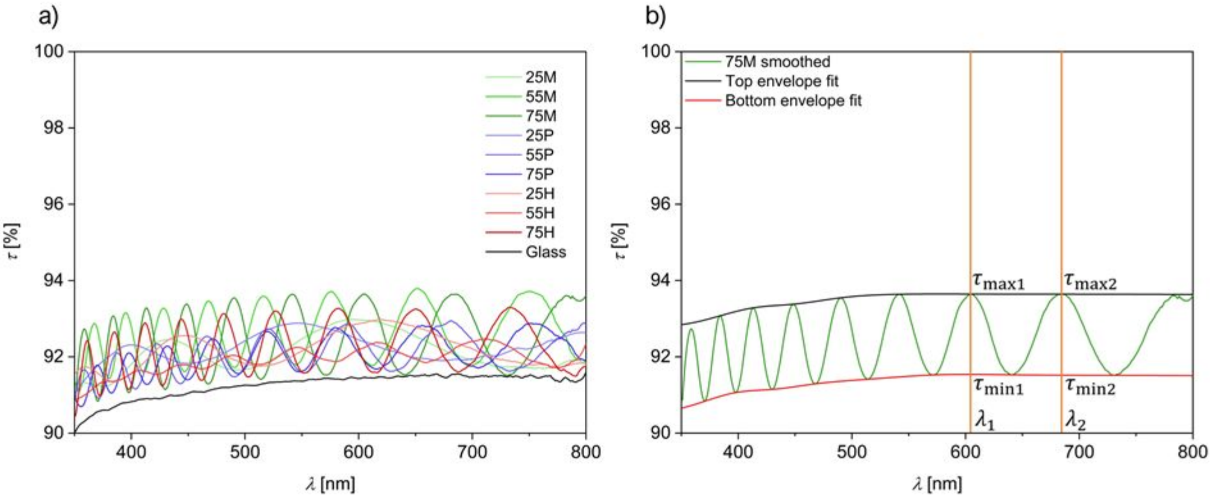

**Figure S3**: a) Smoothed transmission data with the Sav-Gol filter in wavelengths of 350 - 800 nm and b) smoothed 75M transmission spectrum between 350 - 800 nm with top and bottom envelope fits. The two orange lines showcase two points in the transmission spectrum separated by one single oscillation. The marked $\tau$ values represent the transmission point at which the orange lines intercept the envelope fits. The $\lambda$ values represent the point at which the orange lines and thus the maxima of the oscillation intercept the wavelength axis.

**Table S1**: Results of the best fits for the refractive indicies at two wavelengths and the thickness of each coating using the method in ref. [1] and [2].

| Coating | $n_{c1}$ | $n_{c2}$ | $d$ [µm] |
|---|---|---|---|
| 25M | 1.56 | 1.55 | 0.52 |
| 55M | 1.59 | 1.58 | 1.61 |
| 75M | 1.59 | 1.59 | 1.58 |
| 25P | 1.55 | 1.53 | 0.51 |
| 55P | 1.56 | 1.56 | 1.43 |
| 75P | 1.56 | 1.55 | 1.68 |
| 25H | 1.55 | 1.55 | 0.52 |
| 55H | 1.54 | 1.53 | 1.57 |
| 75H | 1.58 | 1.58 | 1.84 |

## Surface Roughness of Coated Samples

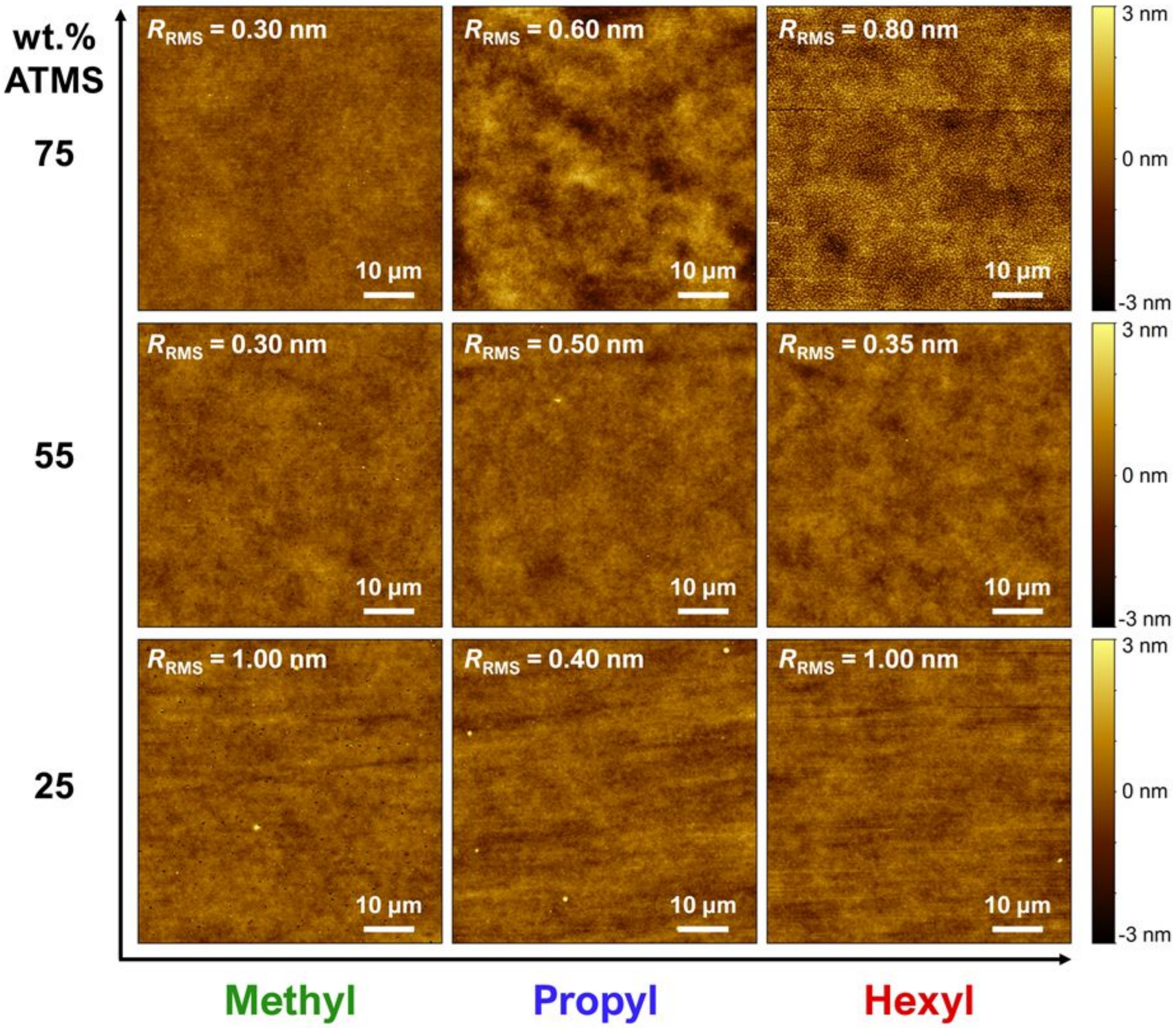


**Figure S4.** AFM topography images ($60 \times 60\ \mu m^2$, $256 \times 256\ px^2$) of silica sol-gel coatings prepared with the three alkyl-TMS series (25, 55, and 75) and different alkyl chain lengths (methyl, propyl, hexyl). All images share the same height scale (±3 nm). The corresponding root-mean-square roughness values ($R_{RMS}$) are indicated in each panel.

The surface topography of the coated samples was analyzed by atomic force microscopy (AFM) in (soft) tapping mode (Dimension Icon, Bruker) using OTESPA cantilevers (≈ 300 kHz, 26 N $m^{-1}$). For each coating, multiple areas ($60 \times 60\ \mu m^2$) were scanned. Polynomial background subtraction (third order) was applied to correct for sample tilt and scanner drift, and the root-mean-square roughness ($R_{RMS}$) was calculated from the leveled height data (Gwyddion software).

Representative topography images are shown in Figure S4. Across all formulations, $R_{RMS}$ values ranged from ~0.3 nm to 1.4 nm. No systematic trend in roughness was observed as a function of alkyl chain length (M, P, H) or alkyl-TMS content (25, 55, 75). The coatings exhibited uniformly smooth and homogeneous surfaces.

# Scanning Drop Friction Force Microscopy

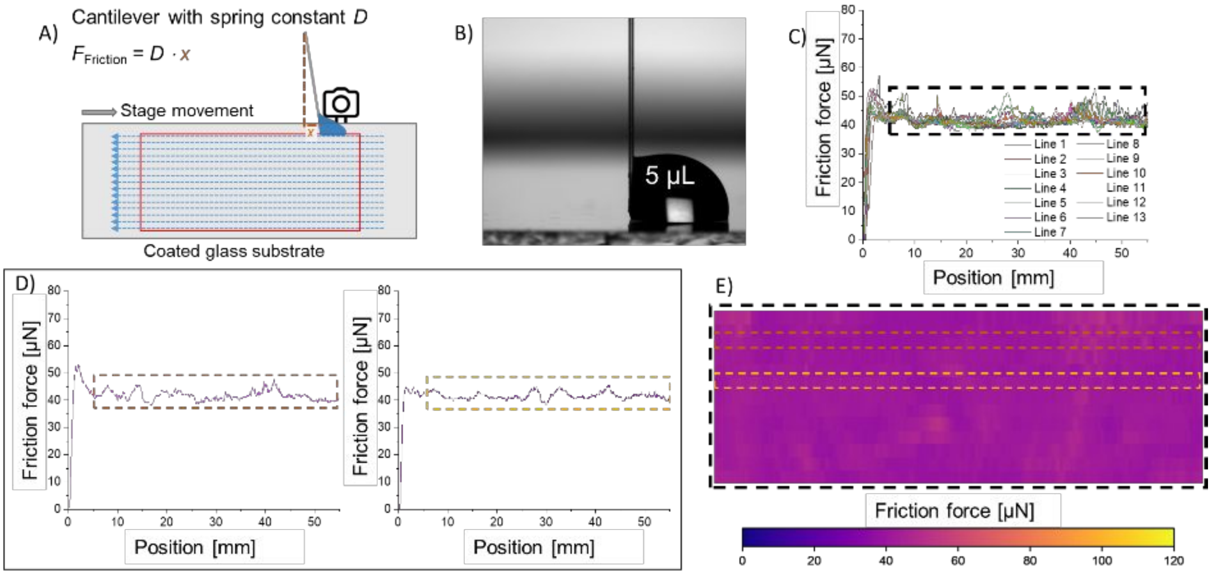


**Figure S5.** (A) Scanning drop friction force microscopy setup. (B) Camera frame of droplet and capillary during scanning. (C) Lateral force traces from individual scan lines. (D) Two representative traces showing the steady sliding segment. (E) Reconstructed friction heatmap.

Here, we illustrate how the camera-recorded capillary deflection is converted to one-dimensional lateral force traces and then to a spatial friction map. The measurement principle (**Figure S5.A**) is described in the main text (**Figure 3b**). A representative camera frame showing the droplet attached to the glass capillary, with the capillary bending as the substrate is translated (**Figure S5.B**). Lateral force–position profiles extracted from each scan line are shown in (**Figure S5.C**). Each curve corresponds to one independent scan across the coating, and the overlap of the traces indicates the uniformity of the sliding response across the measured region. Two example traces highlighting the steady sliding regime are shown in (**Figure S5.D**). The friction force values from all scan lines are assembled into the friction heatmap in (**Figure S5.E**), where each horizontal band corresponds to one scan line.

From the heat maps, histograms of the local kinetic friction forces were generated for each coating (**Figure S6**). These histograms were normalized and fitted with Gaussian functions to extract the mean kinetic friction force and standard deviation for each formulation. The width of the distribution reflects the heterogeneity of droplet mobility across the coating.

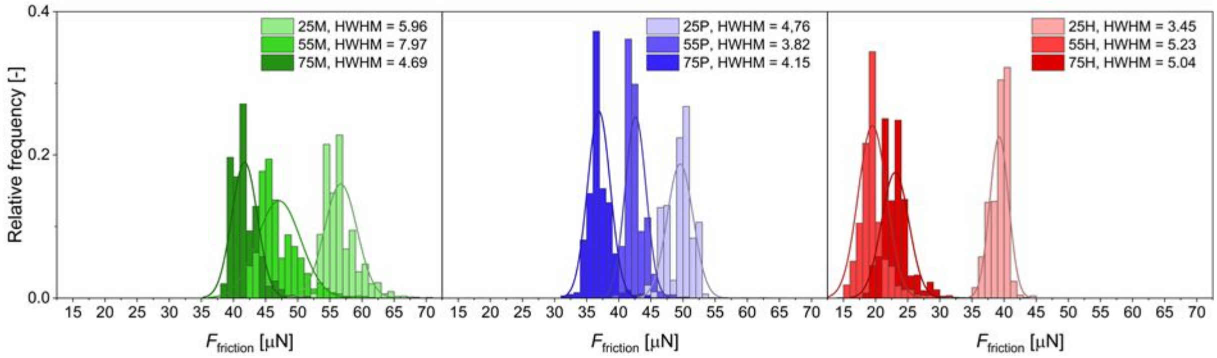


**Figure S6.** Histograms of friction forces obtained from Scanning drop friction force microscopy measurements for each coating.

# Particle size for colloidal probe AFM measurements

The size of the Si-particle used for all colloidal probe AFM measurements was determined with a scanning electron microscope (SEM). Figure S7 shows an SEM image of the particle glued to the tipless cantilever. The nominal size of the particle is 10 µm. We used the SEM images to determine the actual particle radius of 4.74 µm.

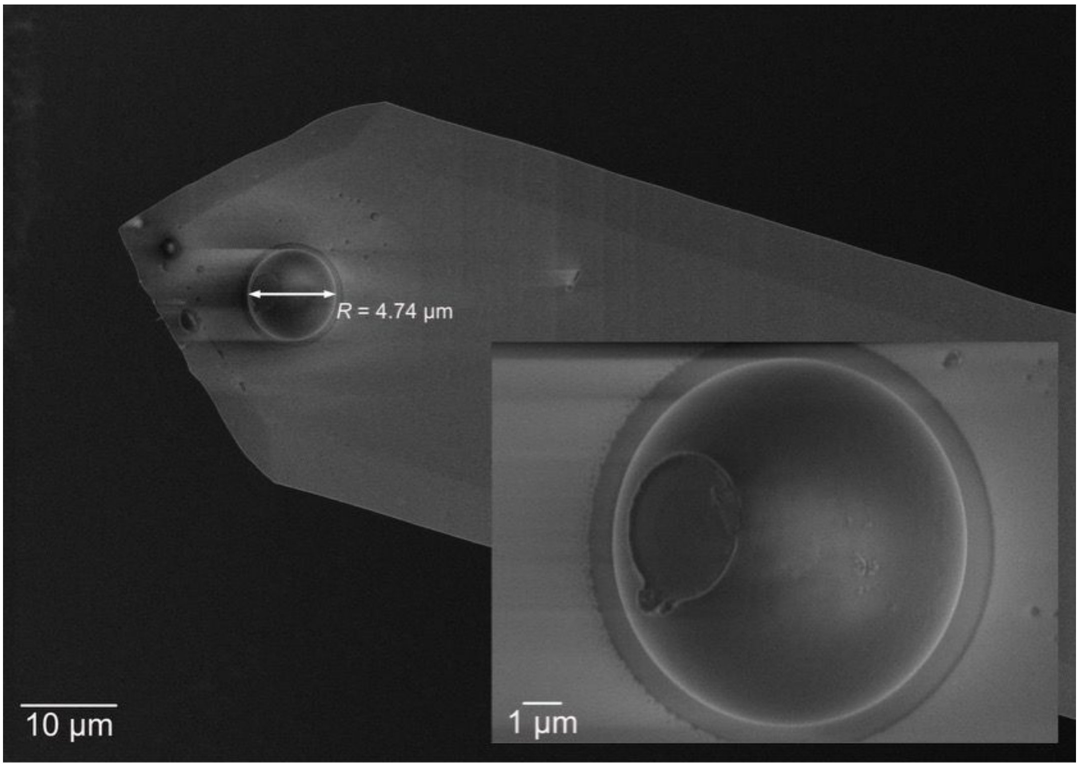


**Figure S7.** SEM image of the particle glued on the tipless cantilever. The nominal particle diameter was 10 μm ($R$ = 5μm). Based on the SEM images we determined a particle radius of 4.7 μm.

# Frequency distributions for determining the mean adhesion force

The mean adhesion force of all alkyl-TMS:TEOS coatings is determined by fitting the frequency distributions of the measured adhesion forces of each coating with a Gaussian fit. **Figure S8** (a) – (c) shows the frequency distributions and the corresponding Gaussian fit for the methyl-, propyl- and hexyl-TMS containing coatings. Based on the Gaussian fit, the mean adhesion force $\overline{F}_{\mathrm{adh}}$ and the error $\Delta\overline{F}_{\mathrm{adh}}$ (see **Figure 5**) are determined by the position of the centre of the fit and 2σ, respectively.

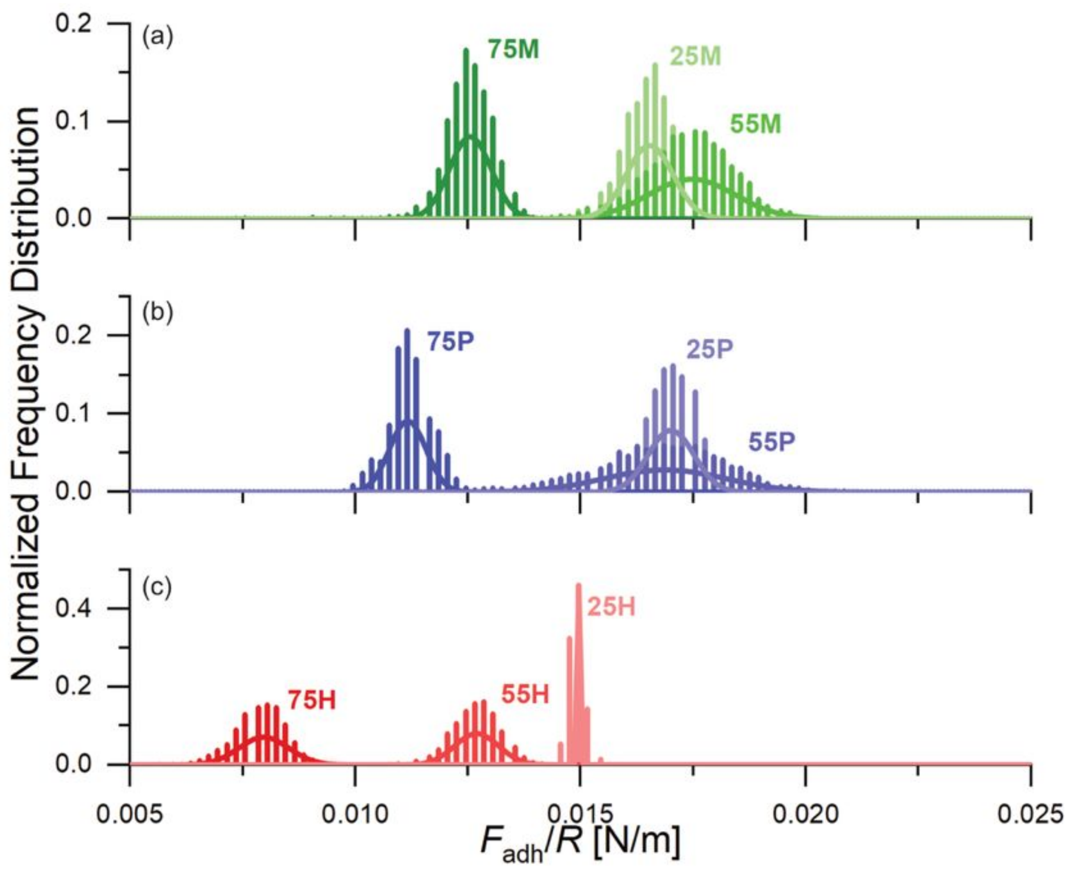


**Figure S8.** Normalized frequency distributions with corresponding curve of the Gaussian fit of the adhesion force for the coatings containing methyl- (a), propyl- (b) and hexyl-TMS (c).

# Further analysis of the adhesion force of the 55P coating

The frequency distribution of the adhesion force of the 55P coating is in comparison to all our other alkyl-TMS:TEOS coatings broader (see **Figure S8** (b)). This results in a larger error of the mean adhesion force, as shown in **Figure 5** (b), and raises the question of whether this in an indication of surface inhomogeneities or changes in the adhesion force during the measurement. Therefore, the adhesion force of the three consecutive 40 x 40 $\mu m^2$ maps (32 x 32 $px^2$ grid) of the 55P coating are shown in **Figure S9** (a) – (c). None of these three maps show distinguishable surface areas or distinct patters that would indicate surface inhomogeneities. Furthermore, the adhesion force does not change between the three consecutive maps, which is additionally illustrated by the corresponding frequency distributions in **Figure S9** (d) – (f). Therefore, the broader frequency distribution of the adhesion force of the 55P cannot be explained by pronounced surface inhomogeneities or changes in the adhesion force during the measurement of consecutive maps.

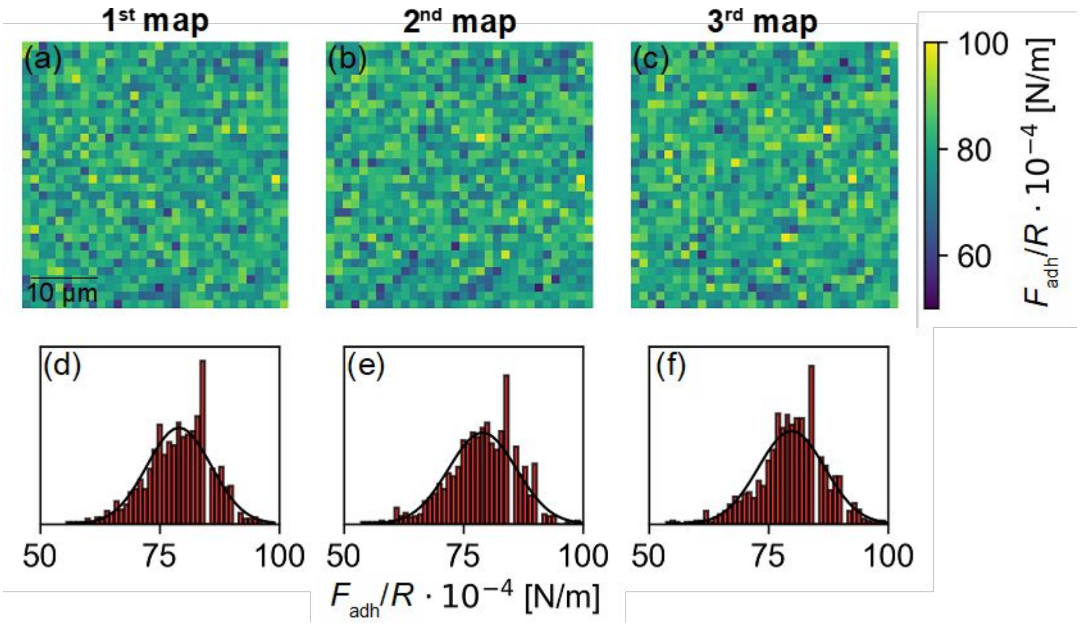


**Figure S9.** (a) – (c) Three consecutive 40 x 40 μm$^2$ maps of the adhesion force of the 55P coating. Note that the same colour scale is used for all three maps. (d) – (f) The corresponding frequency distributions of the adhesion force.

# Adhesion forces at extended contact time

To test whether the contact time between the Si-particle and the underlying coating has an impact on the mean adhesion force, we recorded also force curves with an extended contact time of 125 ms in comparison to 0 ms. Except for the contact time the measurement procedure was the same as described before. A representative baseline-corrected force-time curve (see **Figure S10**) can be divided in three parts: (1) approach of the Si-particle with linear increase in force as soon as the particle and surface are in contact (black), (2) contact is maintained for 125 ms (blue), and (3) withdrawal of the Si-particle with a linear decrease in force until the adhesion force is overcome (red).

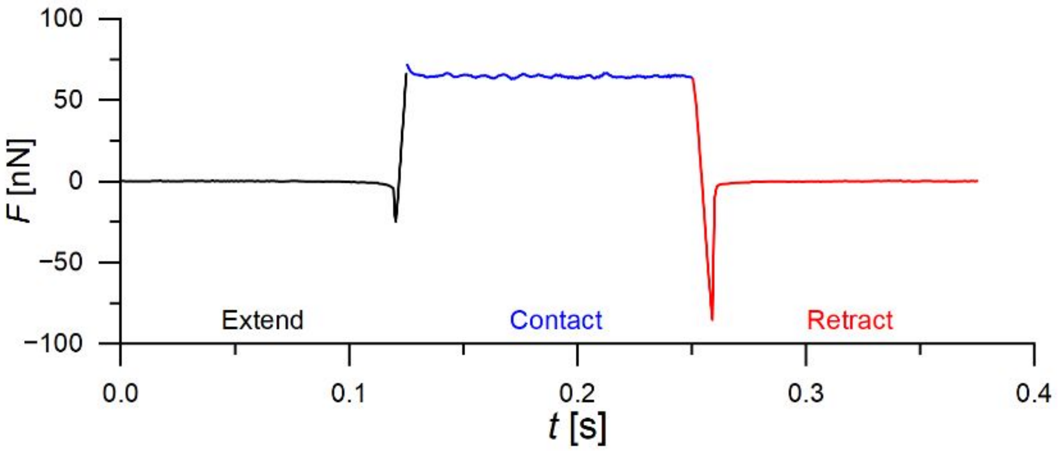

**Figure S10.** Representative baseline-corrected force-time curve for the measurements with the enhanced contact time of 125 ms with highlighted parts showing the approach of the Si-particle (black), the maintenance of contact between Si-particle and surface (blue) and the withdrawal of the particle (red).

The determination of the mean adhesion force for all our alkyl-TMS:TEOS coatings is following the same procedure as described before. Therefore, the frequency distributions are shown in **Figure S11** (a) – (c) for the coatings containing methyl-, propyl-, and hexyl-TMS respectively. In contrast to the frequency distributions at 0 ms contact time (see **Figure S7**), it is noticeable that none of our coatings at a contact time of 125 ms has a comparably broad distribution as the 55P coating at 0 ms contact time.

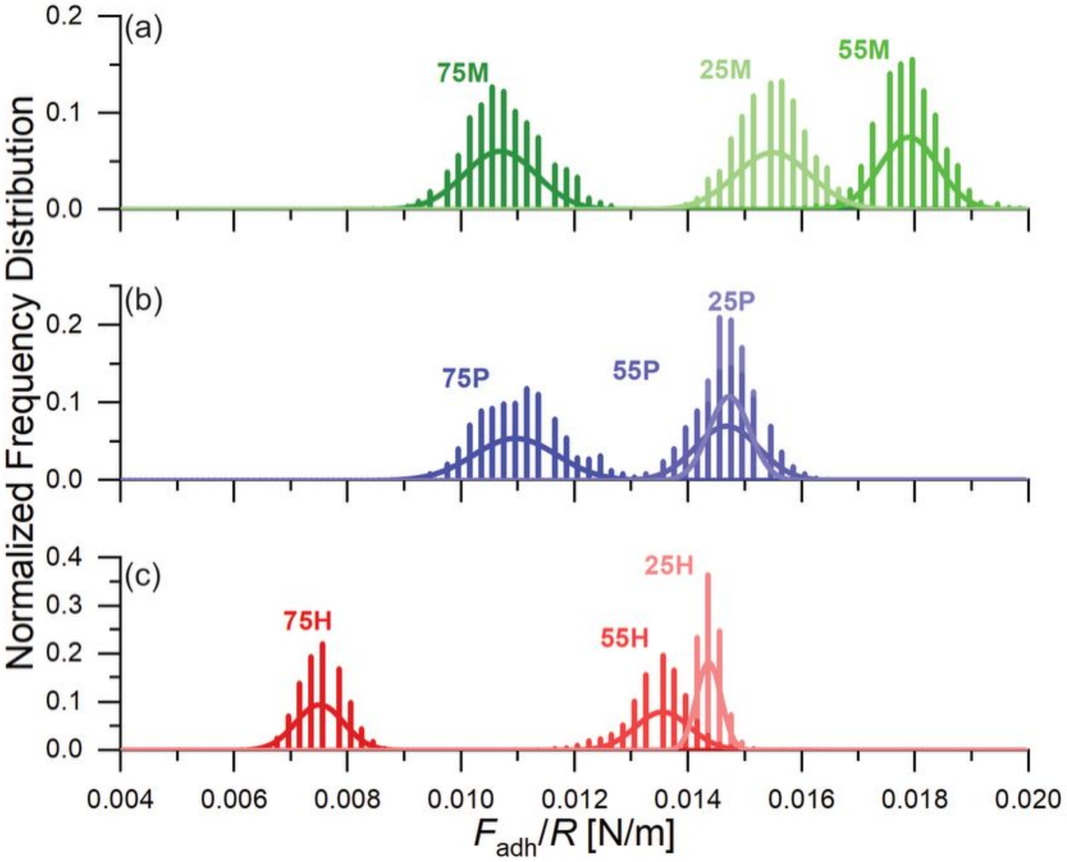


**Figure S11.** Normalized frequency distributions with corresponding curve of the Gaussian fit of the adhesion force for the coatings containing methyl- (a), propyl- (b) and hexyl-TMS (c).

Based on the Gaussian fit of the frequency distributions, the mean adhesion force $\overline{F}_{\mathrm{adh}}$ and the error $\Delta\overline{F}_{\mathrm{adh}}$ (see **Figure S12**) are determined by the position of the centre of the fit and $2\sigma$ respectively. Comparing the mean adhesion forces with and without (see Figure 5 (b)) extended

contact time shows that the mean adhesion force for the hexyl-TMS:TEOS coatings are in agreement regardless of the amount of hexyl-TMS. In contrast to the hexyl-TMS:TEOS coatings, changes in the mean adhesion forces are observed in the methyl- and propyl-TMS containing coatings. The mean adhesion forces of 25M and 25P as well as 55P are lowered by 0.015 N/m at 125 ms contact time. As a result, these mean adhesion forces at an extended contact time are comparable with the mean adhesion force of the 25H coating. In addition, the mean adhesion force of 75M decreases also by 0.011 N/m at 125 ms contact time and is therefore in agreement with the mean adhesion force of the 75P coating. Except for 5M, that does not follow the trend of decreasing with increasing contact time, the methyl- and propyl-TMS coatings show at extended contact time similar mean adhesion forces. Therefore, based on the mean adhesion force it seems to have no impact whether particles should be removed from a methyl- or propyl-TMS containing coating.

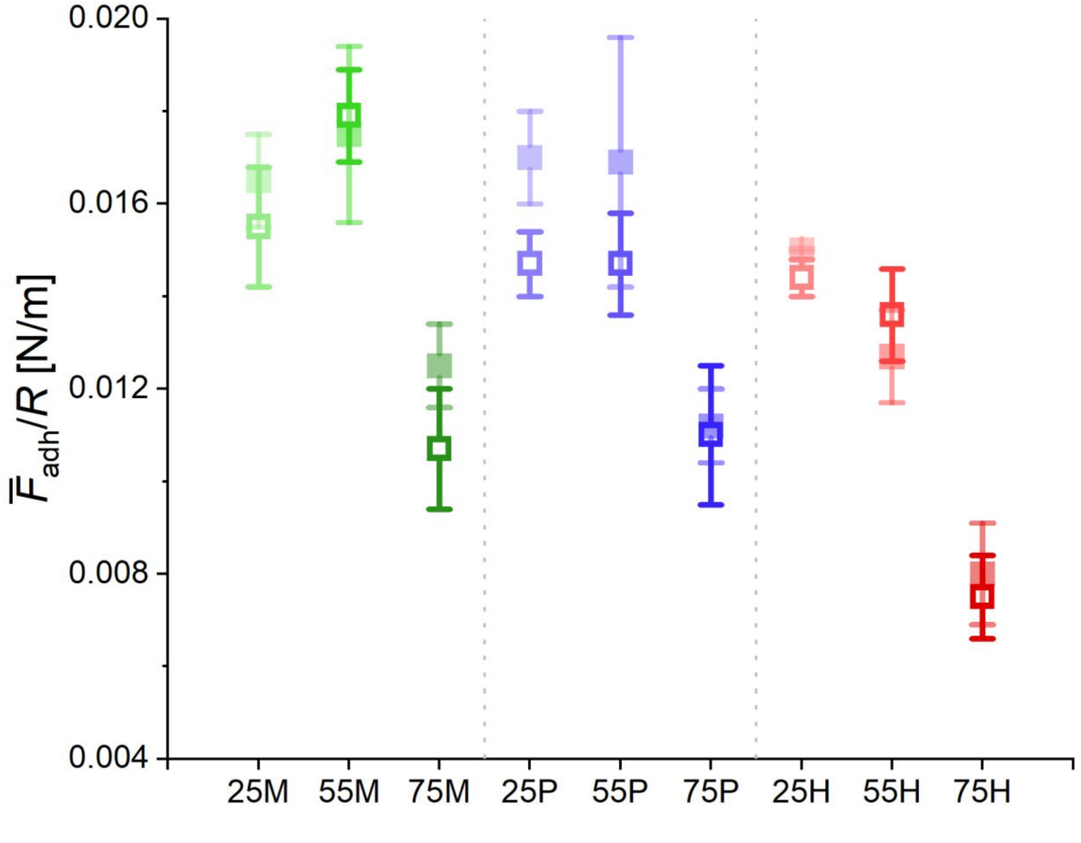


**Figure S12.** Mean adhesion forces between the alkyl-TMS:TEOS coatings and the Si-particle after staying in contact with the surface for 125 ms (open symbols) and for 0 ms (filled, semi-transparent symbols). Coatings containing methyl-TMS are shown in green, propyl-TMS in blue and hexyl-TMS in red, each with the different alkyl-TMS -series (25, 55, and 75).

# Cleaning Tests

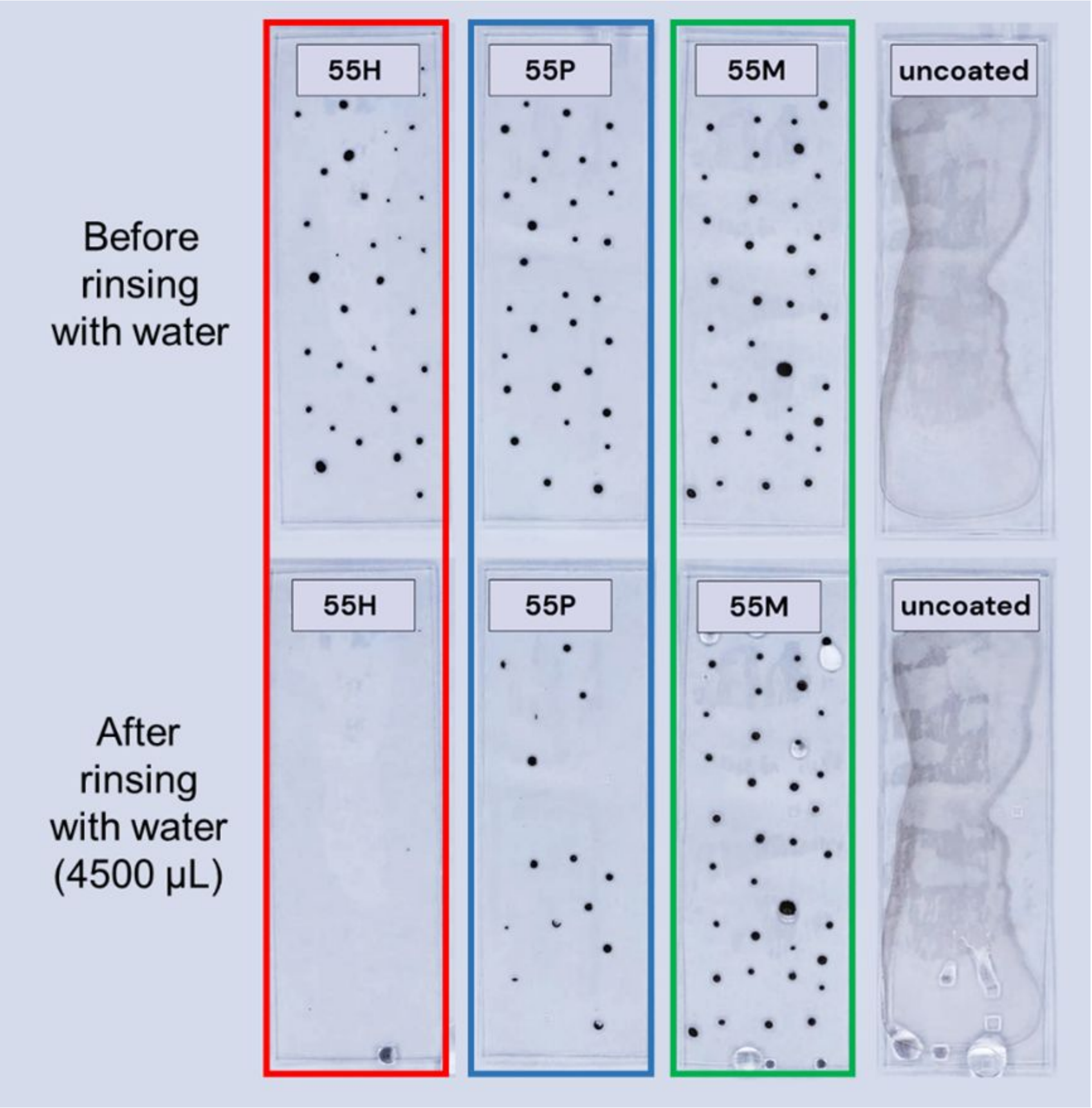


**Figure S13.** Cleaning performance of selected coatings and uncoated glass. Top row: surfaces after uniform artificial soiling (0 µL water applied). Bottom row: same surfaces after application of 4,500 µL water. Coatings shown are 55H (red outline), 55P (blue outline), 55M (green outline), and uncoated soda–lime glass. The 55H coating is fully cleaned under the applied conditions, 55P retains partial contamination, and 55M shows no improvement. The uncoated glass also shows no effective removal of soiling; however, the appearance differs due to its higher hydrophilicity, which alters water spreading and particle distribution.

The cleaning performance of selected coatings was evaluated by applying a controlled volume of water to artificially soiled surfaces. Representative images before (0 µL) and after (4,500 µL) water application are shown in Figure S13. The initial soiling was distributed uniformly across the

coating surface. For the 55H sample, a total of 4,500 µL was sufficient to completely remove all visible contamination. Under identical conditions, the 55P coating exhibited partial cleaning, with some soiling particles remaining on the surface. In contrast, the 55M coating showed negligible change after the same water volume. The uncoated glass also showed no effective particle removal; however, the appearance of the soiling differs due to the much higher hydrophilicity of the bare substrate, which affects the wetting and spreading of water and thus the visual distribution of particles. These results highlight the superior cleaning efficiency of coatings with longer alkyl chains (hexyl-TMS) at higher concentrations, consistent with their lower drop friction and reduced particle adhesion forces. Shorter alkyl chains (propyl- and methyl-TMS) result in less effective removal under the same cleaning conditions, correlating with the trends observed in the friction and adhesion measurements. The corresponding cleaning videos are provided in the supplementary video files.

# Determination of the penetration depth

We define the contact point ($z_0$, $d_0$) as the minimum force point of the extend force vs. z-piezo displacement curve (see Figure S14 (a)). The indentation $\delta$ is calculated by $\delta = (z - z_0) - (d - d_0)$. The resulting force vs. indentation curve is shown in Figure S14 (b). Both curves can be divided in two parts: (1) until the cantilever tip is in contact with the surface ($z$ <2.38µm and $\delta$ <0) and (2) while the cantilever tip is in contact with the surface ($z$ >=2.34µm and $\delta$ >=0). As the cantilever tip is in contact with the surface, two different slopes are visible in the force vs. z-piezo displacement curve (highlighted in blue and orange). This indicates that the upper layer is softer, *i.e.* has a lower elastic modulus, and the layer underneath is harder, *i.e.* has a higher elastic modulus. While the cantilever tip indents the upper layer by ~1 nm, no further indentation is observed for the layer below. All our samples show a similar behavior (see Figure S15). We define the maximum indentation as penetration depth. The averaged penetration depth for each of our samples is shown in Figure 6 (a).

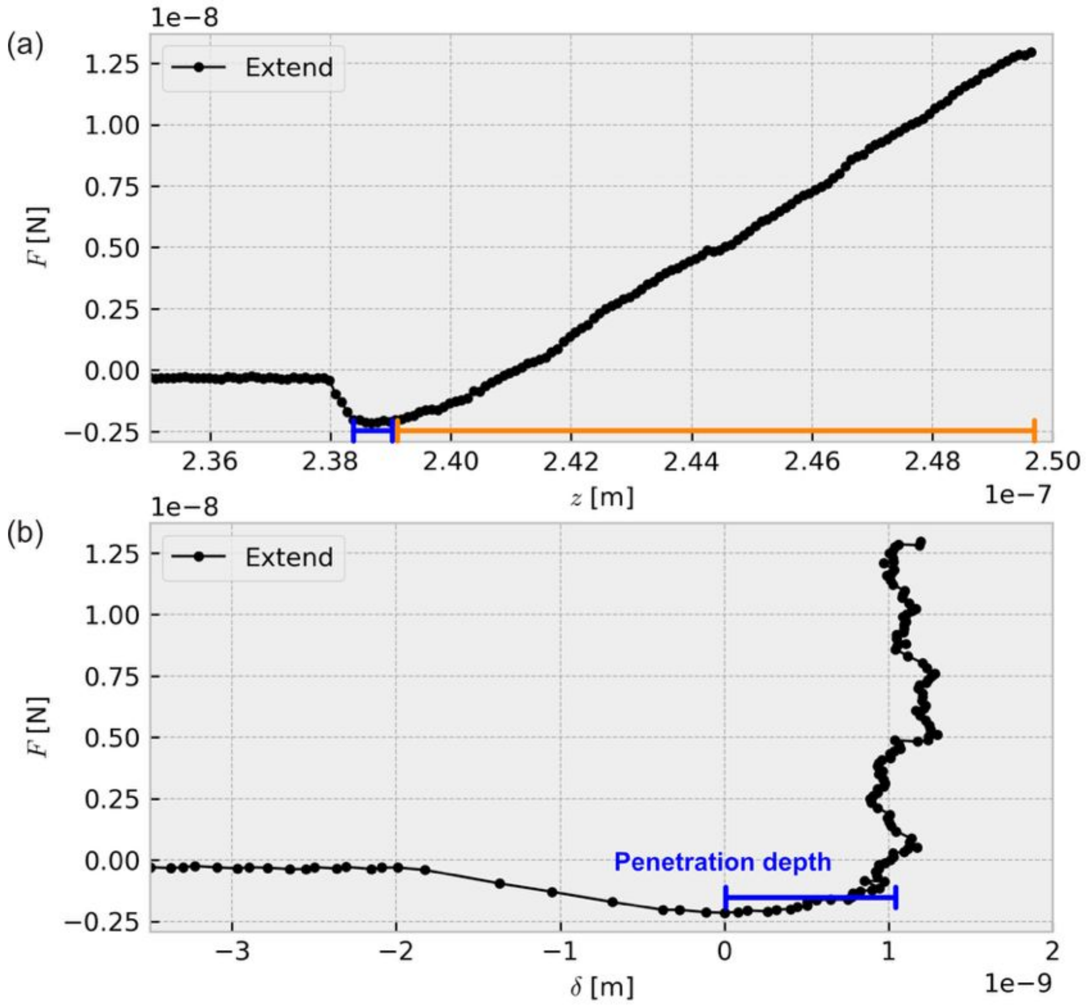


**Figure S14.** Exemplary baseline-corrected extend force F vs. z-piezo displacement curve (a) and force vs. indentation \delta curve (b). While the cantilever tip is in contact with the surface, two different slopes (highlighted in blue and orange) indicate an upper soft layer and a harder layer underneath. While the cantilever tip indents the upper layer by ~1nm (shown in blue as penetration depth), no further indentation is observed in case of the harder layer underneath.

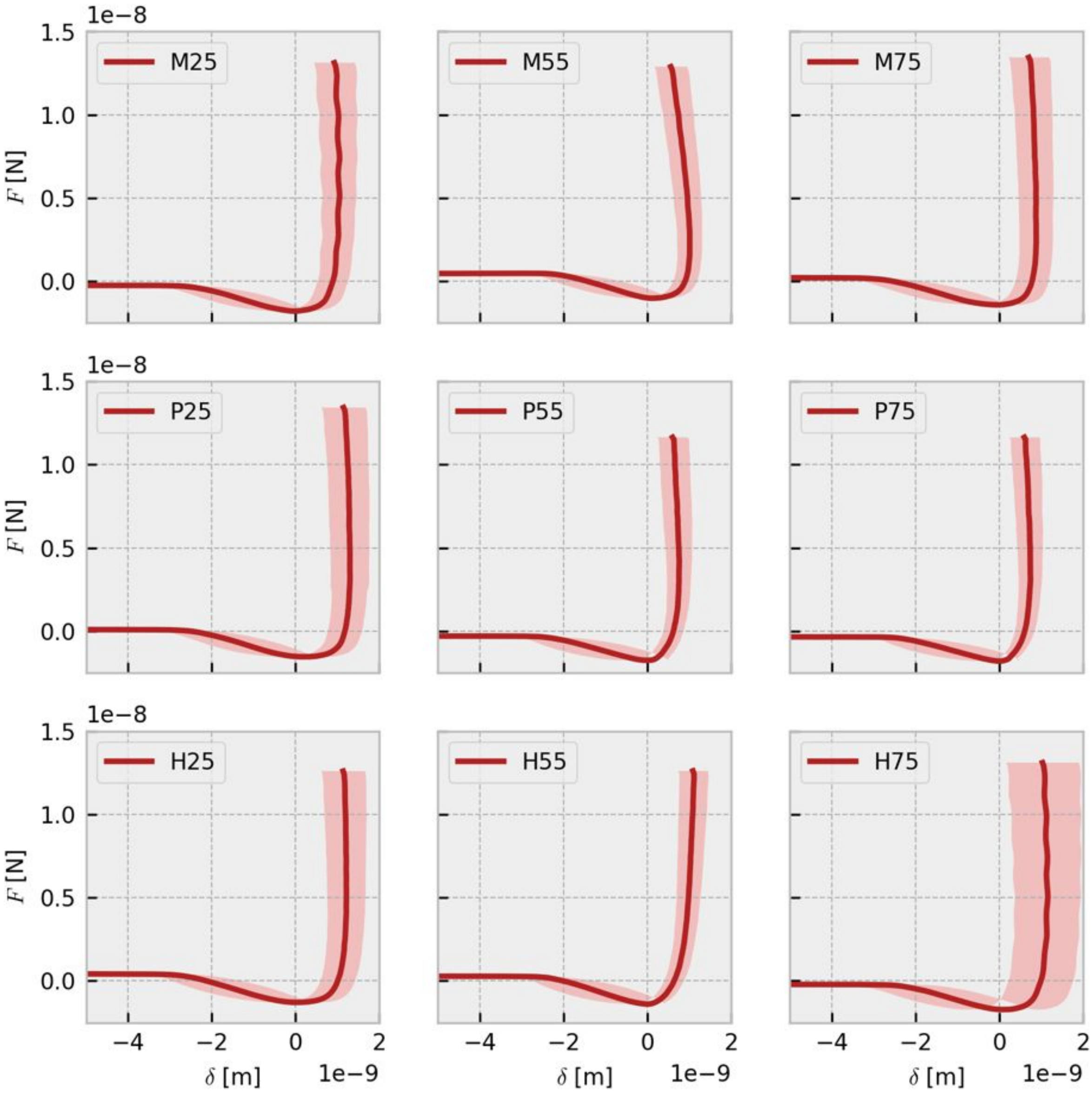


**Figure S15.** Averaged baseline-corrected force vs. indentation curves for all our samples. The standard deviations are displayed semi-transparently. The sample names are given in the upper left corner.

## References


[1] Swanepoel, R. Determination of the Thickness and Optical Constants of Amorphous Silicon. *J. Phys. E: Sci. Instrum.* **1983**, *16* (12), 1214–1222. https://doi.org/10.1088/0022-3735/16/12/023.

[2] Manifacier, J. C.; Gasiot, J.; Fillard, J. P. A Simple Method for the Determination of the Optical Constants n, k and the Thickness of a Weakly Absorbing Thin Film. *J. Phys. E: Sci. Instrum.* **1976**, *9* (11), 1002–1004. https://doi.org/10.1088/0022-3735/9/11/032.
